# Freeze structuring unlocks minimal-processing strategies for legume texturization

**DOI:** 10.1038/s41538-026-00991-5

**Published:** 2026-09-01

**Authors:** Andrea Bach, Elin Perler, Lenja S. Lemcke, Patrick A. Rühs

**Affiliations:** Institute of Food, Nutrition, and Health, Department of Health Sciences and Technology, ETH Zürich, Zürich, Switzerland

**Keywords:** Engineering, Materials science, Plant sciences

## Abstract

Legumes are sustainable, nutrient-rich, and affordable, yet their potential remains underused because conventional food processing often fails to utilize the entire raw material or achieve desirable textures. Here, we demonstrate that whole-legume suspensions can be structured into gels with increased firmness and a distinct anisotropic architecture using directional freezing. The method leverages directional ice crystal growth to exclude legume components from the advancing ice phase, thereby redistributing them within aligned interstitial regions. Upon thawing, these concentrated domains interlink to form a continuous network, reinforcing the gel and generating anisotropic textures. This approach was successfully applied to legumes representing 96% of global production, including chickpeas, soybeans, peas, lentils, lupins, common beans, and mung beans, underscoring its versatility. By eliminating fractionation and additive use, freeze structuring offers a decentralized, sustainable solution for producing nutritious, texturized plant-based foods using standard freezing technologies, accessible for industrial and home-scale production worldwide.

## Introduction

The global food system is at a crucial turning point in adopting sustainable practices to address environmental degradation, resource scarcity, and the growing demand for safe and nutritious food^1^. Plant-based protein alternatives have emerged as a promising solution, offering both environmental and health benefits. Although their popularity increased rapidly over the past decade, recent market slowdowns highlight persistent challenges, including limited consumer acceptance driven by concerns over taste, cost, and excessive processing^2^.

Among plant-based protein sources, legumes stand out as a widely recognized opportunity to advance both sustainability and nutrition. They are nutrient-dense, have a low environmental footprint, and serve as key ingredients in many plant-based products. Their ecological adaptability enables cultivation across diverse agro-climatic zones, supported by extensive genetic diversity that facilitates integration into local food systems worldwide^3,4^. Legumes provide affordable protein and essential micronutrients such as iron, zinc, and vitamins, making them particularly valuable in low- and middle-income countries where animal protein is scarce, and nutrient deficiencies remain widespread. Beyond addressing global malnutrition, legumes are also gaining relevance in high-income countries amid growing interest in vegetarian diets and alternative proteins. Despite these advantages, legume consumption remains constrained by cultural habits, sensory preferences, and socio-economic barriers^5^.

Food processing can play a critical role in addressing these challenges by improving taste, safety, convenience, nutritional quality, and functional performance, thereby expanding the range of legume-based foods^5,6^. However, many existing legume-based products rely on highly refined ingredients and intensive processing, contributing to consumer scepticism associated with perceptions of ultra-processed foods as overly engineered or unnatural^2,7^. Limited access to advanced processing technologies in some regions further restricts adoption. Together, these factors hinder the broader integration of legumes into diverse diets, highlighting the need for innovative approaches that use minimally refined ingredients while preserving desirable sensory and nutritional properties^5,8^.

While processing can enable desirable textures and functionality, dominant texturization technologies such as extrusion present notable limitations. High-moisture extrusion enables controlled development of protein microstructure under regulated thermomechanical conditions^9^. However, it typically relies on protein isolates or concentrates, complex supply chains, substantial capital investment, and tightly controlled processing windows. Its performance is often sensitive to raw material variability, particularly when working with minimally refined ingredients. Dry extrusion is more tolerant of less refined raw materials but frequently struggles to deliver comparable fibrous textures and remains largely inaccessible to consumers^8^. Fermentation approaches, such as tempeh production, can enhance nutritional quality by reducing antinutrients and improving digestibility, yet pose food safety and scalability challenges in decentralized or low-tech settings^11^. Consequently, there remains a need for broadly accessible and safe processing strategies that operate directly on minimally refined ingredients while providing controllable textural outcomes.

Freeze structuring is an emerging processing technology with roots in traditional products such as kōri tofu in Japan and modern commercial applications like Quorn. Freeze structuring, also referred to as ice templating, describes processes in which growing ice crystals during freezing exclude and concentrate solutes and dispersed components into interstitial regions, generating organized porous or fibrous structures^12,13^. When combined with an imposed temperature gradient, as in directional freezing, this mechanism can be guided to form aligned, anisotropic architectures. Related ice-templating approaches are applied across diverse material systems, for example in freeze casting, where directional solidification is combined with solvent removal to produce dry porous scaffolds with controlled anisotropic architectures^14,15^, and in cryogelation, where ice crystals act as temporary porogens while polymer networks form in the unfrozen phase^16,17^. Long recognized for its ability to influence food structure without excessive heat, directional freezing has recently regained attention for creating fibrous, meat-like textures in plant-based products^14–23^. Despite this potential, the broader adoption of freeze structuring has been limited by the complexity of food matrices, comprising proteins, polysaccharides, and lipids that interact across multiple length scales and undergo major structural changes during freezing^18^. As a result, freeze structuring for food has predominantly been carried out using purified ingredients, such as protein concentrates and isolates^19–25^, or specialized equipment^19,23,25^. Advancing freeze structuring beyond refined ingredients toward whole-food systems represents a critical opportunity. However, freeze structuring approaches that combine accessibility, scalability, and compatibility with complex food matrices, while preserving nutritional integrity, are still lacking.

Here, we show that whole-legume suspensions can be structured into gels with increased firmness and a distinct anisotropic architecture using directional freezing alone, without purification, additives, or specialized equipment. By leveraging ice crystal growth to redistribute macromolecular components, this method creates anisotropic, fibrous gels with superior mechanical integrity compared to conventional freezing. Its effectiveness across diverse legumes highlights its potential to democratize legume utilization and enable clean-label, customizable foods. Freezing is widely recognized for extending shelf life, reducing food waste, while preserving nutritional quality, which collectively improves the environmental performance of food systems^26,27^. Freeze structuring leverages these established benefits by using existing cold-chain infrastructure, supporting its use as a practical and resource-efficient texturing strategy for whole-legume foods. By bridging a critical gap in accessible processing technologies, this approach opens new opportunities for sustainable diets and plant-based food innovation.

## Results

### Directional freezing creates anisotropic structure and texture in legume-based systems

**Fig. 1:**
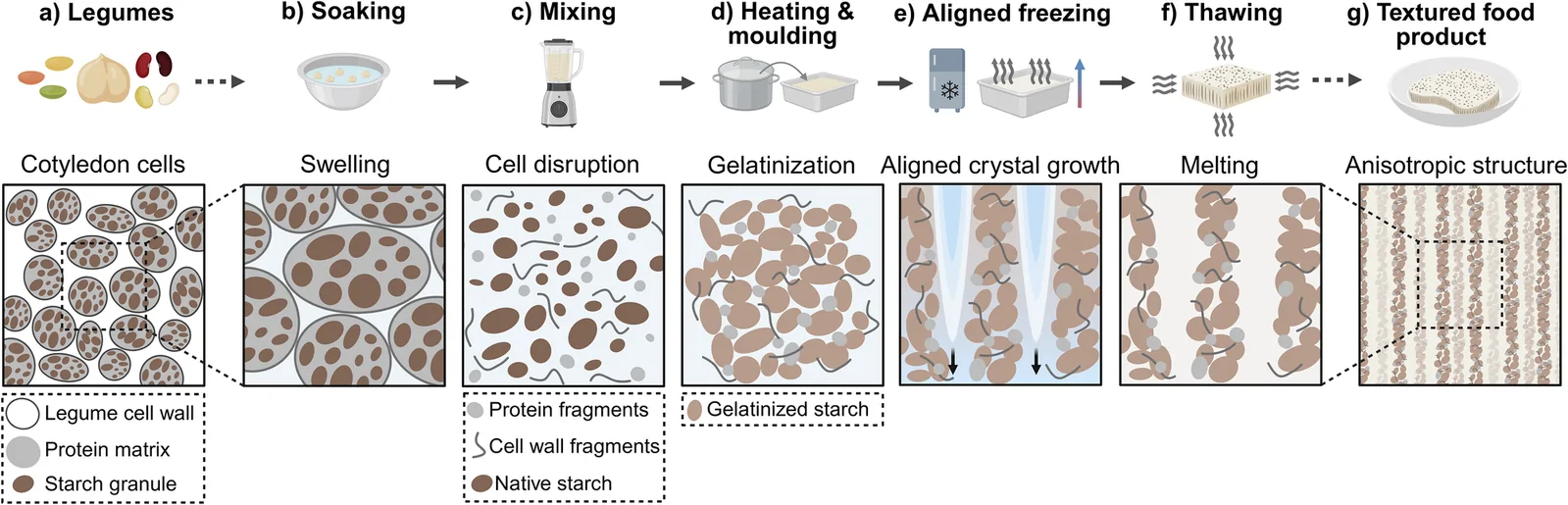
Directional freezing of whole-legume gels creates anisotropic, textured products. **a**, **b** Legume hydration swells cells and starch granules. **c** Mixing disrupts cells and disperses cellular fragments. **d** Heating causes starch gelatinization and protein denaturation, leading to gel formation. **e** Directional freezing concentrates legume components into aligned domains. **f**, **g** After thawing, a lamellar scaffold with self-supporting texture results. Figure inspired by Xiong et al.^28^ and created with BioRender.

Directional freezing of whole-legume gels yields anisotropic, textured food products (Fig. 1). First, legumes are hydrated, causing cotyledon cell swelling, initiating water uptake into starch granules. Mixing disrupts cells and disperses cellular fragments throughout the suspension. Thermal treatment leads to starch gelatinization, protein denaturation, and the simultaneous inactivation of antinutrients^28,29^, producing a gel of different gel strengths depending on the solids content. The molded gel is then subjected to directional freezing, typically by placing it on a cooled surface or insulated in an environment below freezing point, thereby applying a unidirectional temperature gradient^15^. During this step, growing ice crystals exclude suspended legume components from the advancing ice phase, leading to their redistribution within aligned intercrystalline regions^15,30^, reinforcing the gel network^17,31^. After thawing, the ice crystals revert to water, leaving a hydrated, lamellar scaffold that retains the anisotropic alignment formed during freezing and provides a self-supporting texture. Texture development in freeze structuring of cooked legume suspensions is driven by two mechanisms: (1) unidirectional freezing, which imparts alignment, and (2) local concentration increases of legume components through freezing, which enhances gel strength.

### Process conditions define structural outcomes in chickpea gels

**Fig. 2:**
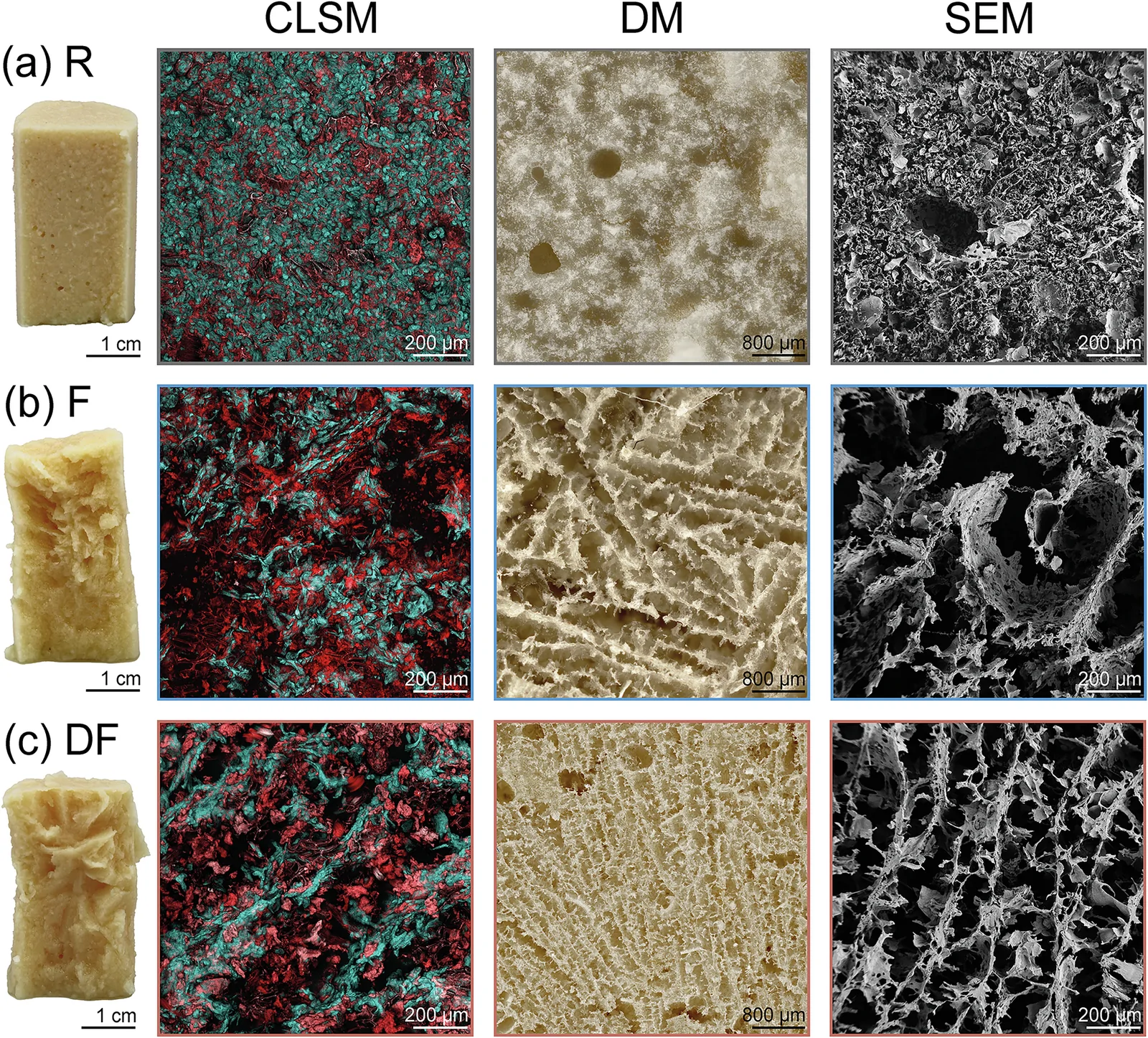
Effect of cooling process on component distribution and microstructure in chickpea-based gels. Photographic images of chickpea gel samples, confocal laser scanning micrographs (CLSM) of chickpea gels stained with FITC (cyan) and rhodamine B (red), digital micrographs (DM), and scanning electron micrographs (SEM) of **a** refrigerated (R), **b** conventionally frozen (F), and **c** directionally frozen (DF) chickpea gels at 12.5 wt%.

Freezing conditions strongly affect microstructural organization and component distribution within chickpea gels, with unidirectional freezing producing the most pronounced structural alignment. To investigate the cooling and freezing conditions that promote anisotropic structuring in 12.5 wt% chickpea gels, three regimes were examined (Fig. 2a–c): (a) refrigeration (R), (b) conventional freezing (F), and (c) directional freezing (DF).

Gels cooled under refrigeration (R) exhibited a homogeneous, isotropic microstructure without visible alignment (Fig. 2a). Cyan-stained spherical domains likely represent swollen starch granules in CLSM (confirmation with iodine staining, S2: Fig. S2), and red fluorescence likely corresponds to cell wall and protein fragments, dispersed throughout the matrix. Subsequent drying preserved this isotropic structure, indicating that the absence of directional thermal gradients maintained the original network.

Conventional freezing (F) produced partial anisotropy, with local alignment but random orientation across the sample (Fig. 2b, orientation analysis, S1: Table S1). Starch granules (cyan) lost their discrete morphology, appearing diffuse and irregular. This is consistent with structural disruption and granule disintegration caused by freeze-thawing after gelatinization, also observed in maize starch systems^32^. Regions of concentrated material alternated with large pores formed by ice crystal growth. After drying, fibrous structures and voids of varying orientation were evident, reflecting mild inward thermal gradients that arise when outer layers solidify faster than the core during conventional freezing, allowing ice crystals to grow rather than nucleate uniformly throughout the sample.

Directional freezing (DF) generated pronounced anisotropy and phase segregation (Fig. 2c orientation analysis, S1: Table S1). Material concentrated along lamellae formed by advancing ice crystals, giving rise to a porous, aligned architecture. Starch-rich regions (cyan) appeared diffuse and were aligned along these lamellae, while protein and cell-wall fragments (red) exhibited less orientation, remaining dispersed between aligned starch domains. After drying, the previously aligned ice regions become clearly visible as lamellar structures interconnected by ridges, a consequence of particle exclusion as ice crystals advanced^33^. DF produced the most distinct structural alignment among all cooling regimes and can also emerge when using simple setups at home (S3 and S4).

### Freezing-induced particle concentration transforms gel properties

**Fig. 3:**
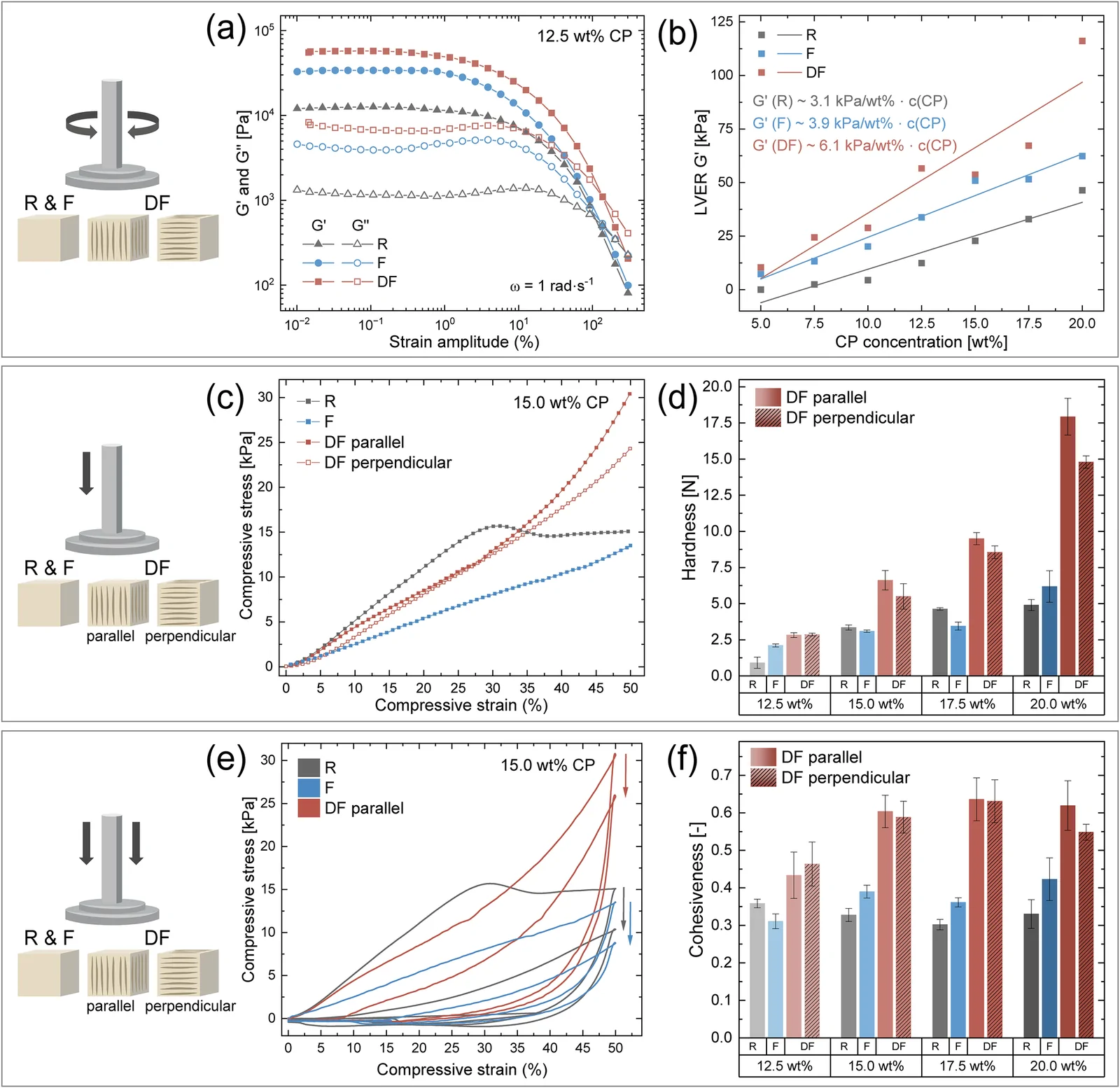
Rheological and mechanical properties of chickpea gels subjected to different cooling and freezing regimes. **a** Oscillatory amplitude sweeps of 12.5 wt% chickpea (CP) gels: refrigerated (R), conventionally frozen (F), and directionally frozen (DF). **b** G′ in the linear viscoelastic region (LVER) of amplitude sweeps of R, F, and DF gels from 5.0 to 20.0 wt%. **c** Stress-strain curves obtained from uniaxial compression of gels at 15.0 wt%, with DF measured parallel and perpendicular to the temperature gradient. **d** Hardness of R, F, and DF gels with concentrations from 12.5 to 20.0 wt%. **e** Stress-strain curves obtained from double compression of gels at 15.0 wt%, with DF measured parallel to the temperature gradient. **f** Cohesiveness of R, F, and DF gels with concentrations from 12.5 to 20.0 wt%.

Since cooling and freeze structuring affect the distribution of chickpea components, the mechanical gel properties were assessed using oscillatory shear rheology and single- and double-compression tests of gels formed by refrigeration (R), conventional freezing (F), and directional freezing (DF) (Fig. 3). DF induces a local increase in the concentration of solid components and promotes the formation of lamellar morphologies, thereby increasing viscoelasticity, hardness, and cohesiveness.

Chickpea suspensions formed viscoelastic gels with predominantly elastic behavior through R, F, and DF. The elastic and viscous moduli (G′ and G″) are higher for F and DF than for R, due to a gel network strengthening effect after thawing of ice crystals, yielding elastic moduli of ~5 × 10⁴ Pa for directionally frozen samples versus ~10⁴ Pa for refrigerated gels formed using 12.5 wt% chickpea suspensions (Fig. 3a). A detailed rheological analysis is provided in the Supporting Information (S5).

Elasticity (G′) scaled linearly with concentration (5.0–20.0 wt%) across all conditions, but the rate of increase varied markedly (Fig. 3b). Refrigerated chickpea gels (R) showed an average linear rise in G’ of 3.1 kPa/wt%, and conventionally frozen gels (F) a rise in G’ of 3.9 kPa/wt%. In contrast, directionally frozen samples (DF) exhibited a ~ 1.5-2x stronger response in elasticity (6.1 kPa/wt%). Above 10.0 wt%, frozen gels were roughly twice as elastic as refrigerated ones, reflecting a higher density of effective crosslinks n (${\rm{G}}^{\prime} \propto {\rm{n}}\cdot {\rm{k}}\cdot {\rm{T}}$). Therefore, freezing can promote gel strength through freeze-concentration effects: as ice crystals grow, water is removed from the system as pure ice, increasing the concentration of solutes and macromolecules in the remaining unfrozen phase and redistributing them within the intercrystalline matrix^15,31^. However, this also reduces the water-holding capacity of the system and leads to syneresis upon thawing, particularly at lower initial concentrations and lower network densities. Despite syneresis, DF gels remained anisotropic and self-supporting, retaining mechanically coherent structures relevant for food applications. Concentrations shown in the graphs refer to the initial prepared suspensions, and elasticity values corrected for syneresis confirm the same trends (regression statistics and syneresis, S6 and S7: Table S2 and S3, Fig. S4).

15.0 wt% chickpea gels exhibited distinct stress-strain responses under R, F, and DF conditions, reflecting differences in network integrity and anisotropic reinforcement induced by the cooling protocol (Fig. 3c). In the initial elastic region (up to ~10.0 % strain), R exhibited the highest slope, followed by DF parallel, DF perpendicular, and then F, indicating that R initially appears stiffer under small deformations. At higher strains, the curves diverge: R reached its maximum stress at ~30% strain, then dipped and plateaued at 15.1 kPa, suggesting structural collapse, whereas DF parallel and perpendicular continued rising to 30.4 kPa and 24.4 kPa, respectively, reflecting sustained strain-hardening and network integrity. F gels showed a consistently lower resistance to deformation and reached a maximum stress of ~13.5 kPa.

Hardness increased nearly linearly with chickpea concentration across all cooling conditions (Fig. 3d), reflecting denser network formation at higher solids content. R and F gels did not differ significantly, whereas DF gels were consistently harder, with values ~ 1.5-3x higher than R and F. DF samples prepared with 15.0 to 20.0 wt% compressed parallel to the freezing direction were harder by factors of 1.1 to 1.2 than those compressed perpendicular, confirming pronounced anisotropy. This directional dependence arises from the lamellar microstructure formed during directional freezing: parallel compression engages structurally reinforced domains, whereas perpendicular compression disrupts weaker interlamellar regions. Corrected comparisons accounting for syneresis confirm that, at equivalent effective concentrations, DF gels remain harder than R and F gels (statistics and syneresis correction, S8 and S9: Tables S4–S11, Fig. S5 and Fig. S6).

DF gels exhibited the highest cohesiveness, the ability of a gel to withstand a second deformation after the first, indicating a more elastic and structurally resilient texture compared to R and F gels (Fig. 3e, f). R, F, and DF chickpea gels at 15.0 wt% indicated structural weakening and permanent deformation upon repeated compression, reflected in a reduced load-bearing capacity during the second deformation (Fig. 3e). Cohesiveness remained relatively constant across concentrations, with slightly lower values at 12.5 wt%, but DF gels were consistently more cohesive than R and F (Fig. 3f), demonstrating that DF gels preserve network connectivity more effectively upon reloading. This suggests a more integrated and elastic internal network in DF gels, while R and F lack the highly aligned, anisotropic architecture needed for efficient load redistribution under repeated strain. DF and R gels maintained high recovery values (≈82-84%) across most concentrations, while F gels had significantly lower springiness (≈68%), indicating that DF gels recover their height after compression similarly to R gels, but with a more robust internal structure (cohesiveness, springiness and statistics, S10–S12: Tables S12–S25, Fig. S7a).

Directionally frozen (DF) chickpea gels exhibit mechanical properties that align closely with those of established protein-based foods while introducing distinctive structural features. Chewiness, which reflects the combined effect of hardness, cohesiveness, and springiness during repeated compression, offers a practical measure of overall bite resistance. The combination of greater hardness and cohesiveness in DF gels resulted in markedly higher chewiness (average of 4.4 N) compared to R and F gels (1.0 and 1.1 N, respectively) (chewiness and statistics, S11 and S13: Fig. S7b, Tables S26–S32). DF chickpea gels spanned 0.9 to 10.0 N across 12.5 to 20.0 wt% formulations. At 20.0 wt%, DF gels reached 8.7–10.0 N, closely matching mozzarella (~9.7 N) and exceeding Brie (~5.7 N), while remaining well below firm tofu (~20.2 N). At lower concentrations (12.5–15.0 wt%), chewiness values fell between 0.9 and 2.9 N, similar to silken tofu (~0.85 N) (references measured under identical double compression conditions, S14: Table S33). This progression highlights the tunability of DF gels, from soft tofu-like textures at low concentrations to cheese-like chewiness at higher concentrations. Unlike many conventional products, DF gels exhibit a uniquely aligned network, characterized by visually distinct lamellar domains (Fig. 2) and hardness anisotropy ratios exceeding 1.1–1.2. In structured foods, such anisotropic organization can enhance structural complexity, a key factor in creating diverse textural experiences and supporting product differentiation^34^. Freeze structuring thus provides a versatile approach for tailoring the texture and mouthfeel of legume-based gels across a wide range of culinary applications.

### Extending freeze-enabled texturization to diverse legumes

**Fig. 4:**
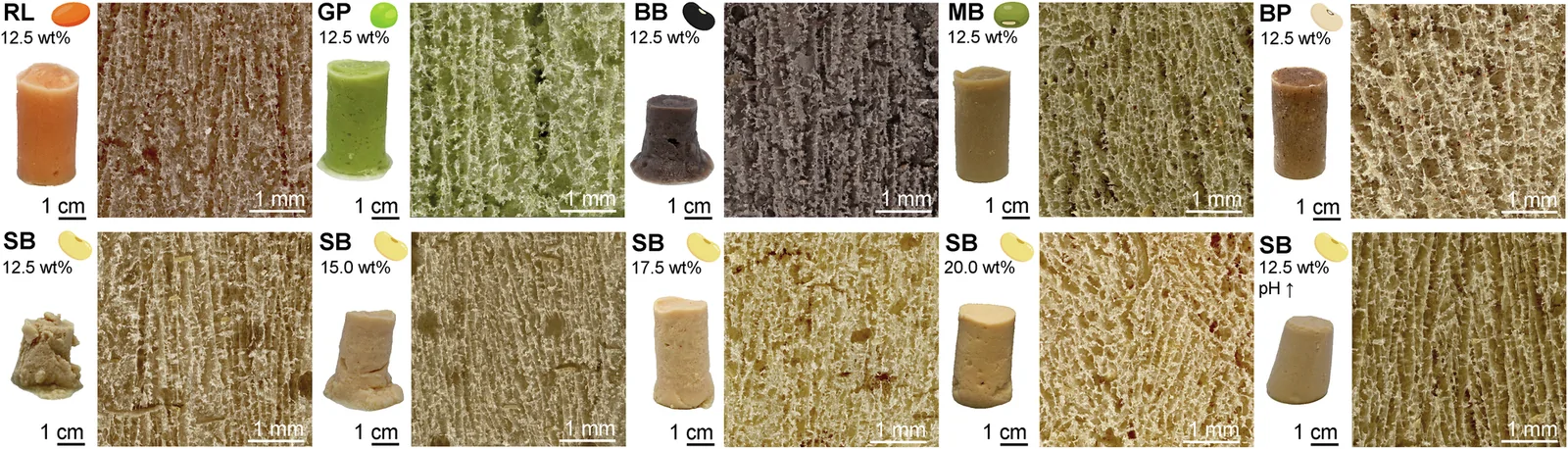
Directionally frozen legume gels. Photographic images of gels prepared from whole red lentils (RL), green peas (GP), black beans (BB), mung beans (MB), black-eyed pea (BP) and soybeans (SB) at 12.5 wt% solids after thermal treatment and directional freezing. Additional samples include soybean (SB) gels at elevated concentrations (15.0 to 20.0 wt%) and pH-adjusted SB gels (pH 10.4).

To assess the broader applicability of freeze-induced structuring of minimally processed legumes, we extended the approach beyond chickpeas to legumes representing 96 % of the global production^35^ (worldwide legume production, S15: Table S34). Gels made from whole red lentils (RL), green peas (GP), black beans (BB), mung bean (MB), black-eyed peas (BP), and soybeans (SB) were thermally treated to produce gels of various strength depending on solids content and legume type and were subsequently subjected to directional freezing and assessed for their structural alignment and textural properties. The resulting samples revealed apparent differences in textural stability and visual anisotropy (Fig. 4).

All tested legumes formed anisotropically structured gels at 12.5 wt% solids, confirming the broad applicability of freeze-induced alignment (Fig. 4). However, mechanical integrity varied among legume species. Red lentil (RL), black lentil (BL), and mung bean (MB) produced firm, stable gels with minimal syneresis, whereas black bean (BB) yielded slightly softer gels. Soybean (SB) gels at 12.5 wt% had low gel stability but still exhibited pronounced lamellar alignment under directional freezing. The textural properties are likely attributable to the elevated lipid content in soybeans, which can disrupt starch network formation and compromise overall gel integrity (SB composition, S17: Table S35). Increasing the solids concentration to 15.0 and 17.5 wt% improved SB texture while maintaining clear structural anisotropy. At 20.0 wt%, mechanical strength further increased. However, the visual definition of anisotropic domains became less pronounced, likely due to reduced availability of freezable water at high solids content and thus limited ice crystal growth and phase segregation during freezing^33^. Furthermore, pH increase (pH 10.4) prior to heat treatment enhanced gel firmness at 12.5 wt%, with higher pH likely promoting protein unfolding, highlighting the role of protein solubility, particle size, and surface hydrophobicity in textural outcomes^36^. Other legumes, including black lentils, yellow lentils, kidney beans, navy beans, and lupins were also successfully anisotropically structured under DF (S16: Fig. S8).

Gel strength appeared to increase with decreasing protein/non-fiber-carbohydrate (g/g) ratio of legumes (legume composition, S17: Table S35). Samples with relatively low ratios (e.g., red lentil 0.52, green pea 0.59, mung bean 0.42, black-eyed pea 0.48) formed self-supporting gels with higher mechanical integrity, likely due to a higher proportion of non-fiber carbohydrates, including starch, which promotes starch gelatinization and formation of a continuous load-bearing network. In contrast, samples with relatively high protein/non-fiber-carbohydrate ratios, such as soybean (1.85), exhibited weaker gel formation, likely due to limited starch content and protein dominance, which can hinder starch gelatinization and disrupt network continuity. Similar effects have been reported in mixed pea protein-maize starch systems, where increasing protein fractions weakened starch-dominated gel networks^37^. In addition to compositional ratios, starch-specific properties, including granule size, swelling capacity, and amylose to amylopectin ratio, may further modulate gel strength and network organisation, contributing to the observed variability among legumes^37,38^.

These findings confirm that freeze structuring is broadly applicable across legumes and textural outcomes are strongly influenced by intrinsic composition. Key parameters such as solids concentration, particle size, and protein solubility can be strategically adjusted to tailor mechanical properties without compromising structural anisotropy. Further customization can be achieved by formulating mixtures of legumes with varying gel-forming capacities. Alternatively, process adaptations, such as modifying gelation, homogenization, or freezing conditions, offer further opportunities to fine-tune structure and texture. Directional freezing enables the development of functional, anisotropic textures without reliance on refinement or purification steps, thereby preserving dietary fiber and other nutritionally and functionally valuable components present in the starting raw material. In contrast, conventional plant-based processing typically relies on fractionation and refinement to achieve functional products. For example, tofu production requires the separation of okara, resulting in substantial macronutrient redistribution^39^: approximately 85% of dietary fiber, 16% of lipids, and 23% of protein from whole soybeans are removed into okara and whey (compositions, S18, Table S36). Similarly, extrusion-based processes commonly involve ingredient fractionation to obtain functional protein or starch fractions^10^. These steps, although integral to those technologies, can result in nutrient losses and generate side streams^8,10^. By avoiding fractionation and refinement, freeze structuring maintains the whole-ingredient composition, which supports more resource-efficient and sustainable food processing^8^.

## Discussion

Freeze structuring through directional freezing provides a simple and robust strategy to generate anisotropic, fibrous textures in minimally processed whole-legume gels without the need for purification or additives. By redistributing macromolecular components and inducing lamellar architectures, the process transforms whole-legume suspensions into mechanically reinforced gels with distinct anisotropic structure, as confirmed by microscopic, rheological, and mechanical analyses. Compared with conventional freezing or refrigeration, directionally frozen systems consistently exhibit higher mechanical strength and directional dependence of texture. The successful application across multiple legumes, including chickpeas, lentils, peas, and various beans, highlights the versatility of this approach and its capacity to produce diverse textural outcomes from a variety of raw materials.

Beyond its functional benefits, freeze structuring offers a processing route that operates directly on minimally processed legumes using simple freezing protocols compatible with existing cold-chain infrastructure. At the household scale, the process can be carried out using standard domestic freezers without requiring specialized equipment. At small- and medium-enterprise levels, the method is compatible with commonly used freezing technologies, as it relies only on conventional thermal treatment and freezing steps. In contrast to conventional plant-based structuring technologies that rely on ingredient fractionation and refinement, directional freezing organizes naturally present macromolecules within whole-legume suspensions into anisotropic architectures. This ability to generate texture directly from minimally processed legume materials may offer sustainability advantages by reducing ingredient isolation and recombination, although dedicated comparative assessments are needed to quantify these potential benefits. While prior research has primarily focused on purified protein or polysaccharide systems, this work demonstrates that directional freezing can effectively structure unrefined, thermally treated legume suspensions into fibrous gels with tunable mechanical properties. Together, these findings indicate that freeze structuring is applicable across multiple legume types and processing conditions, highlighting its potential for broader use.

Future work should systematically investigate how functional properties of legume components, such as protein gelation and starch gelatinization behavior, and formulation conditions such as pH, influence structure formation during directional freezing and the resulting mechanical properties, as well as potential implications for protein digestibility and nutrient bioaccessibility. Translation to consumer and culinary applications will benefit from the development of simple, reproducible preparation protocols that integrate soaking, milling, thermal treatment, and freezing using standard kitchen equipment. The structuring mechanism remains effective in the presence of spices and herbs, enabling flavor customization without compromising texture. Providing clear procedural guidance alongside ingredient flexibility may facilitate the adoption of freeze structuring in everyday practice and support its potential contribution to sustainable, clean-label and minimally processed food systems.

## Methods

Commercial chickpeas (*Cicer arietinum*) were soaked in excess tap water for 24 h at 4 °C. After soaking, the pulses were drained and diluted with water to achieve a dry matter content of 20.0% (w/w), then blended into a homogeneous slurry using a Vitamix Ascent A3500i blender (Vitamix, Ohio, USA). This stock was further diluted to working concentrations of 5.0, 7.5, 10.0, 12.5, 15.0, and 17.5% (w/w). Aliquots of 5-20 wt.%(50 mL) were transferred into Falcon tubes and heated in a water bath (Caso SV900 Sous Vide Gare, Caso Design, Arnsberg, Germany) at 90 °C for 30 min to induce thermal gelation. Samples were inverted every 30 s during the first 10 min and gently inverted every 5 min thereafter to ensure uniform thermal treatment.

Three post-gelation conditions were directly applied: Refrigeration (R): Samples were stored at 4 °C for 25 h in 50 mL falcon tubes. Conventional Freezing (F): Samples were pre-cooled at 4 °C for 1 h, then frozen in a shock freezer at −15 °C for 24 h (Hugentobler FrigoJet 5 × 1/1 GN, Schönbühl, Switzerland). Directional freezing (DF): Samples were poured into custom insulated silicone molds (S3: Fig. S3a and b), pre-cooled at 4 °C for 1 h, and frozen unidirectionally at −15 °C for 24 h in a shock freezer. On the bottom of each mold (56 mm height; outer diameter 74 mm; inner diameter 28 mm), an aluminium foil was added to prevent low-viscosity samples from running out. Then, the mold was placed on a 10 mm-thick aluminium plate to create a bottom-up temperature gradient and covered with a 21 mm-thick silicone lid after filling (S3: Fig. S2a). This configuration ensures directional solidification from the aluminium interface upward, resulting in anisotropic structuring (S3: Fig. S2b). The temperature profiles of the DF and F samples, as well as the aluminum plate temperature and the shock-freezer air temperature, are provided in the Supporting Information (S19: Fig. S9). Similar results could be achieved using simple setups, such as placing the molded legume gels in a plastic container with expanded polystyrene foam (e.g., Styrofoam) side insulation, all within a standard household freezer (S3: Fig. S3c, d). A practical home guide for inducing anisotropy is provided (S4).

After freezing, all samples were thawed at room temperature for 4 h prior to analysis. The same procedure was performed on other legumes, including soybeans (*Glycine max*), green peas (*Pisum sativum)*, black-eyed pea (*Vigna unguiculata*), red, black and yellow lentils (*Lens culinaris*), mung beans (*Vigna radiata*), lupins (*Lupinus albus*), and common beans (*Phaseolus vulgaris*) namely black beans, kidney beans, navy beans. For pH-adjusted soybean suspensions, the pH was adjusted to pH 10.4 using 1 M NaOH prior to the heating step.

### Rheology

Amplitude sweeps were performed to characterize the gel strength of heated chickpea suspensions under the three structuring conditions (R, F, DF) using a rheometer (MCR 302, Anton Paar GmbH, Graz, Austria) with a plate-plate geometry (PP25), with both upper and lower plates roughened, and a gap of 1 mm at 25 °C. Strain was increased from 0.01% to 300% at a constant frequency of 1 rad s⁻¹. From the amplitude sweeps, the plateau storage modulus (G′) was calculated as the mean G′ within the linear viscoelastic region (LVER), defined as the strain range in which G′ remained above 95% of its initial value.

### Texture analysis

Texture was evaluated using single- and double-compression tests on a texture analyzer (TA.XTplus, Stable Micro Systems, Surrey, UK) equipped with the *Exponent software* (v6.2.6.0). Samples were equilibrated to room temperature and cut into cubes of 15 mm edge length using a sharp kitchen knife. R, F, and DF chickpea gels at 12.5, 15.0, 17.5, and 20.0 wt% were tested. Texture Profile Analysis (TPA) was performed with a 40 mm flat probe by compressing each sample twice with a 5 kg load cell, with DF measured parallel and perpendicular to the temperature gradient. Pre-test, test, and post-test speeds of 300, 180, and 300 mm min⁻¹ were applied, respectively. Each compression reached 50% of the original sample height, with a 5 s interval between compressions. All measurements were performed in triplicate, using cubes taken from the middle section of each sample’s height. For reference values, the same procedure was used to test commercial Brie cheese, mozzarella, silken tofu, and firm tofu. Hardness was defined as the maximum force during the first compression, springiness as the ratio of sample height recovered between the first and second compression, cohesiveness as the ratio of the positive area of the second compression to that of the first compression, and chewiness as hardness × cohesiveness × springiness^40^.

### Confocal laser scanning microscopy (CLSM)

R, F, and DF gels were cut into thin slices of approximately 1 mm using a razor blade. The CLSM method was adapted from Lyu et al.^37^. A staining solution containing fluorescein isothiocyanate isomer I (FITC, CAS 3326-32-7, Sigma-Aldrich, St. Louis, USA) and rhodamine B (CAS 81-88-9, Sigma-Aldrich, St. Louis, USA) (each at 0.0025 w/v%) in N,N-dimethylformamide (DMF, anhydrous, 99.8%, CAS 68-12-2, Sigma-Aldrich, Germany) was used. Gel slices were incubated in the dye solution for 1 h, followed by three washes with deionized water over a total of 30 min to remove excess dye.

Confocal imaging was performed on a Leica TCS SP8 (Leica Microsystems, Wetzlar, Germany) using a 10× objective (0.32 NA, HC PL FLUOTAR). Z-stacks were acquired for each sample (100 slices, 1 μm spacing) over a representative region. FITC was excited with a 488 nm laser and detected between 500–530 nm. Rhodamine B was excited with a 552 nm laser and detected between 584 and 620 nm. Images were adjusted for brightness and contrast.

### Photographic images

Photographic images were taken using a Canon EOS 70D (Canon Inc., Tokyo, Japan) mounted inside a photobox with constant lighting conditions. Images were adjusted for brightness and contrast.

### Digital Microscopy (DM)

F, and DF gels were cut along the freezing temperature gradient into 3–4 mm slices using a carpet knife and freeze-dried for 24 h in a freeze dryer (FreeZone 4.5 L, Labconco, Kansas City, USA) connected to a rotary vane vacuum pump (117 L min⁻¹, 230 V, Labconco, Kansas City, USA) at −84 °C. For refrigerated gels, a slice of the same dimensions was immersed in liquid nitrogen and subsequently freeze-dried. Samples were imaged using a digital microscope (VHX-X1F, Keyence, Osaka, Japan) with a VH-Z20T lens and side-light adapter. Images were acquired at 100× and 150× magnification, stitched, and adjusted for brightness and contrast.

### Scanning electron microscopy (SEM)

DF and F chickpea gels were cut across the freezing gradient into 3–4 mm slices using a carpet knife and freeze-dried for 24 h in a freeze dryer (FreeZone 4.5 L, Labconco, Kansas City, USA) connected to a rotary vane vacuum pump (117 L min⁻¹, 230 V, Labconco, Kansas City, USA) at −84 °C for SEM imaging. Rapid handling of frozen samples, standardized slice thickness and orientation, and identical drying conditions were applied to ensure comparability across all samples. For refrigerated gels, a slice of the same dimensions was immersed in liquid nitrogen and subsequently freeze-dried. Freeze-dried samples were trimmed into 4–5 mm cubes and mounted on SEM stubs with the cut surface (perpendicular to the temperature gradient) facing upward. A 4 nm platinum coating was applied using a compact coating unit (CCU-010, Safematic GmbH, Switzerland). Imaging was performed on a GeminiSEM 450 (Carl Zeiss Microscopy GmbH, Jena, Germany) equipped with an SE2 detector, using an acceleration voltage of 2.00 kV and an aperture current of 100 pA. The working distance was adjusted individually to obtain optimal focus, resulting in values of 8.3 mm (F), 8.8 mm (DF), and 9.5 mm (R), and all micrographs were acquired at a magnification of 50×.

### Iodine staining

Heat-induced molecular changes of chickpea (CP) suspensions were examined with light microscopy. Starch was stained with Lugol’s solution prepared following the protocol using elemental iodine (I2, resublimed, VWR chemicals, Japan) and potassium iodide (KI, >99.5%, Honeywell Fluka, Germany), dissolved in deionized water at a mass ratio of 1:2 (I2:KI)^41^. Changes of CP suspensions upon heat treatment were evaluated using 10 g of heat-treated (30 min at 90 °C) and non-heat-treated CP suspensions (1.0 wt%), which were stained with one drop (0.03–0.05 g) of Lugol’s solution. The stained solutions were immediately examined under a Leica light microscope (DM6 B, Leica Microsystems GmbH, Wetzlar, Germany).

### Orientation and pore analysis

Microstructural alignment and pore size were quantified from digital microscopy images using Fiji (ImageJ, National Institutes of Health, USA). For both analyses, stitched digital microscopy (DM) images acquired at 20× magnification were converted to 8-bit grayscale and cropped where necessary. Structural orientation was quantified using the Directionality plugin in Fiji applying the Fourier components method, which estimates dominant orientations from the spatial frequency distribution of image features. Orientation histograms were generated over an angular range of 0–180°. For each cooling condition, four images were analysed. Gaussian functions were fitted to the histograms to extract the dominant orientation (direction), dispersion of orientations, relative amount of aligned structures, and goodness of fit.

For pore size analysis, an automatic threshold was applied to generate binary images separating pores from the surrounding matrix. R samples were excluded from this analysis because no visible pores were present. Pore size distributions were determined using the Analyze Particles function in Fiji. To exclude small non-pore structures detected during thresholding, a minimum particle area threshold of 0.05 mm² was applied. For each detected pore, the particle area (mm²) and the minimum Feret diameter (MinFeret, corresponding to the minimum caliper diameter) were extracted. These parameters were used to quantify lamellae spacing and pore size distributions across the analysed images.

### Sample syneresis

Syneresis was measured by placing thawed gel samples on a 0.5 mm mesh sieve for 3 min to collect the drained liquid. The liquid was weighed, and syneresis was expressed as the drained mass relative to the initial sample mass. The gel’s solids concentration was then recalculated by adjusting the initial concentration based on the proportion of liquid lost.

### Temperature profiles

Temperature measurements were obtained using a multichannel temperature logger equipped with thermistor sensors (Testo 176 T3, Baden-Württemberg, Germany). For the DF setup, individual sensors were embedded in silicone molds positioned along the side of the molds at defined heights corresponding to the DF plate (0 cm), DF bottom (0.5 cm), DF middle (2.8 cm), and DF top (5.1 cm). The sensor used to record the air temperature was placed freely in the surrounding air next to the setup without enclosure. Temperature data were recorded every 15 s.

## Supplementary information


Supplementary Information.


## Data Availability

The datasets generated and analyzed during the current study are not publicly available due to the use of commercially sourced raw materials that exhibit batch to batch variability, which limits the broader scientific utility of the specific raw data. Nevertheless, as demonstrated in the study, the method is robust across different legumes and batches and does not depend on the characteristics of any single material. The datasets are available from the corresponding author on reasonable request.

## Code availability

Data analysis was carried out using established software tools (Fiji/ImageJ, OriginPro and Python). The processing steps, parameter settings, and evaluation criteria are detailed in the Methods section.
