# Supplementary material for: Freeze structuring unlocks minimal-processing strategies for legume texturization
Source: NPJ Sci Food. 2026 Sep 1;10:258. doi: 10.1038/s41538-026-00991-5 (PMC13534525; doi:10.1038/s41538-026-00991-5)
Supplement: Supplementary file 1 [file 41538_2026_991_MOESM1_ESM.pdf]

Supporting Information

Freeze structuring unlocks minimal-processing strategies for legume texturization

Andrea Bach, Elin Perler*, Lenja S. Lemcke, and Patrick A. Rühs*

Institute of Food, Nutrition, and Health, Department of Health Sciences and Technology, ETH Zürich

**co-first authors*

S1. Orientation and pore analysis

Microstructural alignment and pore size were quantified from digital microscopy images using Fiji (ImageJ, National Institutes of Health, USA).

Orientation analysis

For directionality analysis, digital microscopy (DM) images acquired at 20× magnification were converted to 8-bit grayscale and cropped where necessary. Structural orientation was quantified using the Directionality plugin in Fiji applying the Fourier components method, which estimates dominant orientations from the spatial frequency distribution of image features. Orientation histograms were generated over an angular range of 0-180°. For each cooling condition, four images were analysed. Gaussian functions were fitted to the histograms to extract the dominant orientation (direction), dispersion of orientations, relative amount of aligned structures, and goodness of fit.

DF samples exhibited a dominant orientation of 91 ± 21°, indicating that pores were preferentially aligned along the vertical (y) direction corresponding to the freezing direction (Table 1). The relatively low dispersion (7 ± 4°) indicates a narrow orientation distribution consistent with anisotropic lamellar structures. The amount parameter was 18 ± 8 %, and the goodness of fit of the Gaussian model was 0.43.

In contrast, F samples showed a mean orientation of 86°, but with a very large standard deviation (77°), indicating that pore orientations were distributed across nearly the full angular range. The dispersion was higher (14 ± 11°) and the amount parameter larger (28 ± 18 %), reflecting broader and less distinct orientation peaks. Directionality analysis was not performed for R samples, as these samples did not contain visible pores and therefore did not permit meaningful orientation analysis.

Table S1. Directionality parameters obtained from Fourier-based orientation analysis of microscopy images. Direction represents the dominant structural orientation, dispersion the angular spread of orientations, amount the relative contribution of aligned structures, and goodness the Gaussian fit quality. Values are mean and standard deviation (n = 4 images).

	Direction (°)	Dispersion (°)	Amount (-)	Goodness (-)
**DF average**	90.800	7.473	0.180	0.428
**DF SD**	21.690	3.886	0.083	0.161
**F average**	86.788	13.798	0.285	0.310
**F SD**	77.851	11.037	0.206	0.152

Pore size analysis

Pore size was quantified from the same microscopy images after identical preprocessing steps (conversion to 8-bit grayscale and cropping when required). An automatic threshold was applied to generate binary images separating pores from the surrounding matrix. R samples were excluded from this analysis because no visible pores were present.

Pore size distributions were determined using the Analyze Particles function in Fiji. To exclude small non-pore structures detected during thresholding, a minimum particle area threshold of 0.05 mm² was applied. For each detected pore, the particle area (mm²) and the minimum Feret diameter (MinFeret, corresponding to the minimum caliper diameter) were extracted. These parameters were used to quantify lamellae spacing and pore size distributions across the analysed images.

Pore size distributions differed between freezing conditions (Figure S1). For pore area (Figure S1a), F samples exhibited larger pores overall, with a higher mean pore area (0.57 ± 0.82 mm²) compared with DF samples (0.23 ± 0.50 mm²). The median values showed the same trend, with F samples reaching 0.185 mm² and DF samples 0.096 mm². Both conditions displayed broad distributions with several large outliers, indicating substantial heterogeneity in pore size.

A similar tendency was observed for the minimum caliper diameter (MinFeret) (Figure S1b), which represents the smallest pore dimension. F samples showed larger pore diameters on average (0.66 ± 0.56 mm) than DF samples (0.50 ± 0.44 mm).

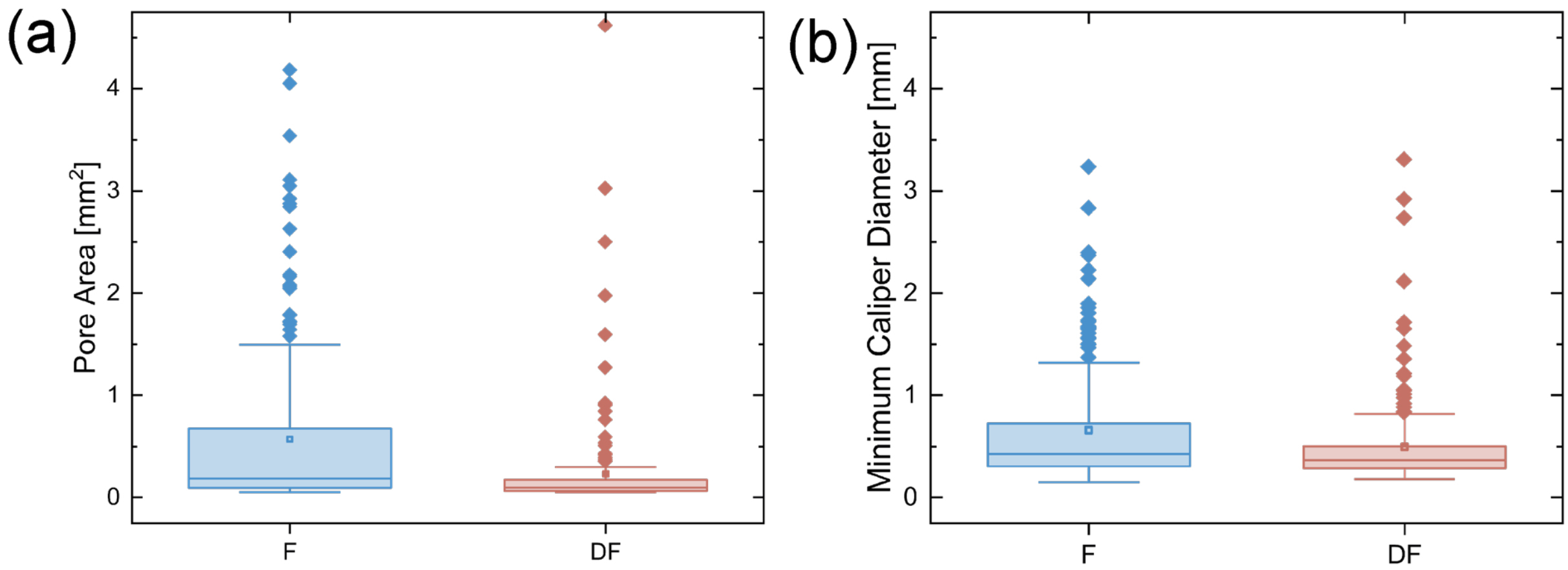

Figure S1. Pore size distributions obtained from microscopy images. (a) Pore area (mm²) and (b) minimum Feret diameter (minimum caliper diameter, mm) for conventional freezing (F) and directional freezing (DF). Boxes represent the interquartile range, center lines the median, squares the mean, and whiskers the data range with outliers shown as points.

S2. Iodine staining

Heat-induced molecular changes of chickpea (CP) suspensions were examined with light microscopy. Starch was stained with Lugol's solution prepared following the protocol using elemental iodine (I2, resublimed, VWR chemicals, Japan) and potassium iodide (KI, >99.5%, Honeywell Fluka, Germany), dissolved in deionized water at a mass ratio of 1:2 (I2:KI)[1]. Changes of CP starch upon heat treatment were evaluated using 10 g of heat-treated (30 minutes at 90 °C) and non-heat-treated CP suspensions (1.0 wt%), which were stained with one drop (0.03 - 0.05 g) of Lugol's solution. The stained solutions were immediately examined under a Leica light microscope (DM6 B, Leica Microsystems GmbH, Wetzlar, Germany) (Figure S2).

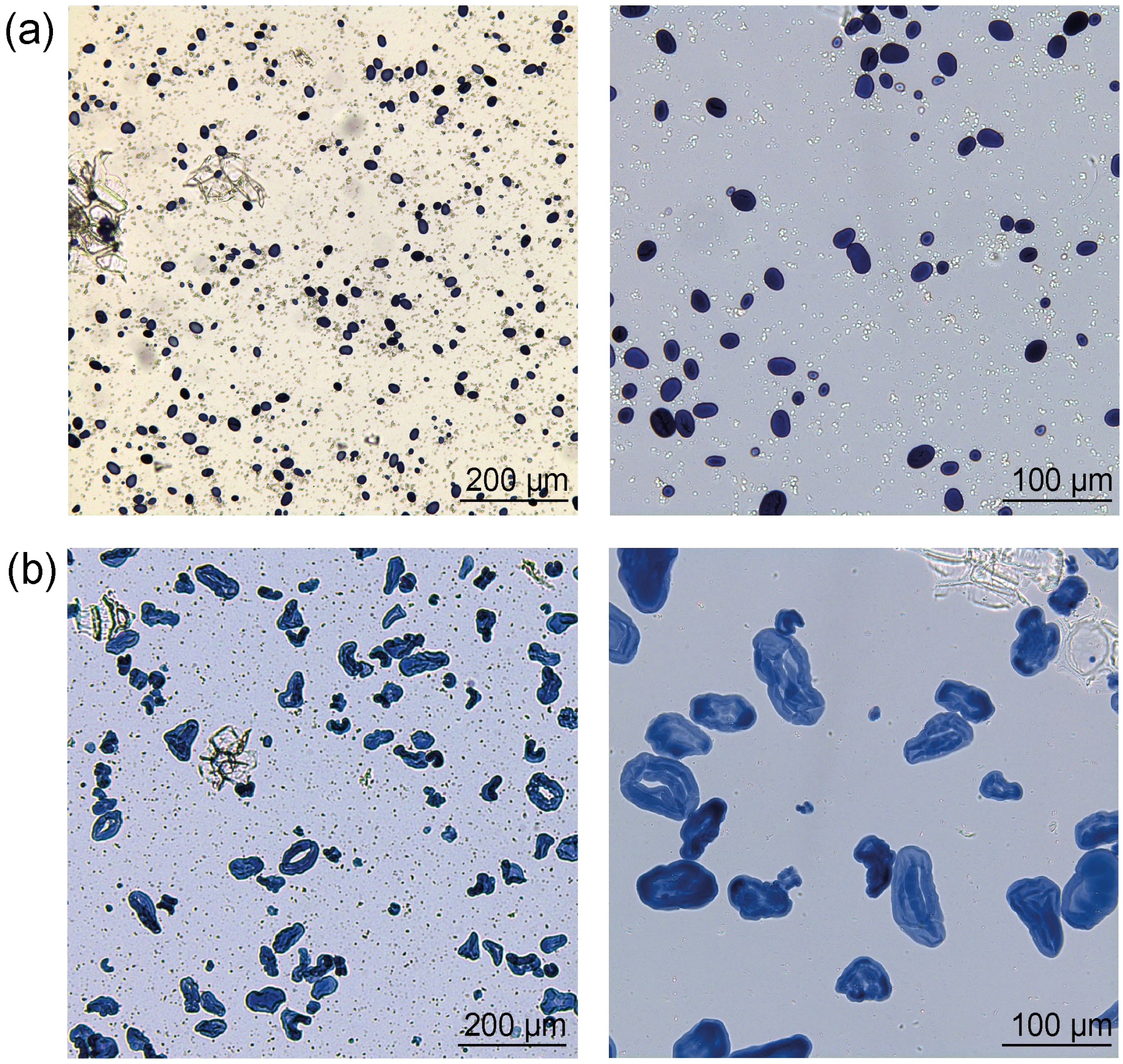

Figure S2. Comparison of chickpea suspensions before and after thermal treatment. (a) 1.0 wt% chickpea suspension stained with iodine solution (pre-heating). (b) 1.0 wt% chickpea suspension after heating at 90 °C for 30 min, stained with iodine solution (post-heating).

S3. Freezing process

Directional freezing can be implemented using different setups by establishing a controlled temperature gradient that drives solidification from one side to the other. Silicone moulds (56 mm height; outer diameter 74 mm; inner diameter 28 mm) were employed for controlled laboratory experiments (Figure S3). On the bottom of each mould, an aluminium foil was added to prevent low-viscosity samples from running out. Then, the mould was placed on a 10 mm-thick aluminium plate to create a bottom-up temperature gradient and covered with a 21 mm-thick silicone lid after filling (Figure 2a). This configuration ensures directional solidification from the aluminium interface upward, resulting in anisotropic structuring (Figure 2b). For simplified, household-scale experiments, a plastic beaker or container, either cylindrical or cuboid, can be inserted snugly into an expanded polystyrene (EPS) foam container (e.g., Styrofoam) to provide insulation and minimize lateral heat transfer (Figure 2c). Beakers with diameters of 3-10 cm and heights of 10-25 cm were successfully used in this setup, which enables anisotropic structuring without specialized equipment (Figure 2d).

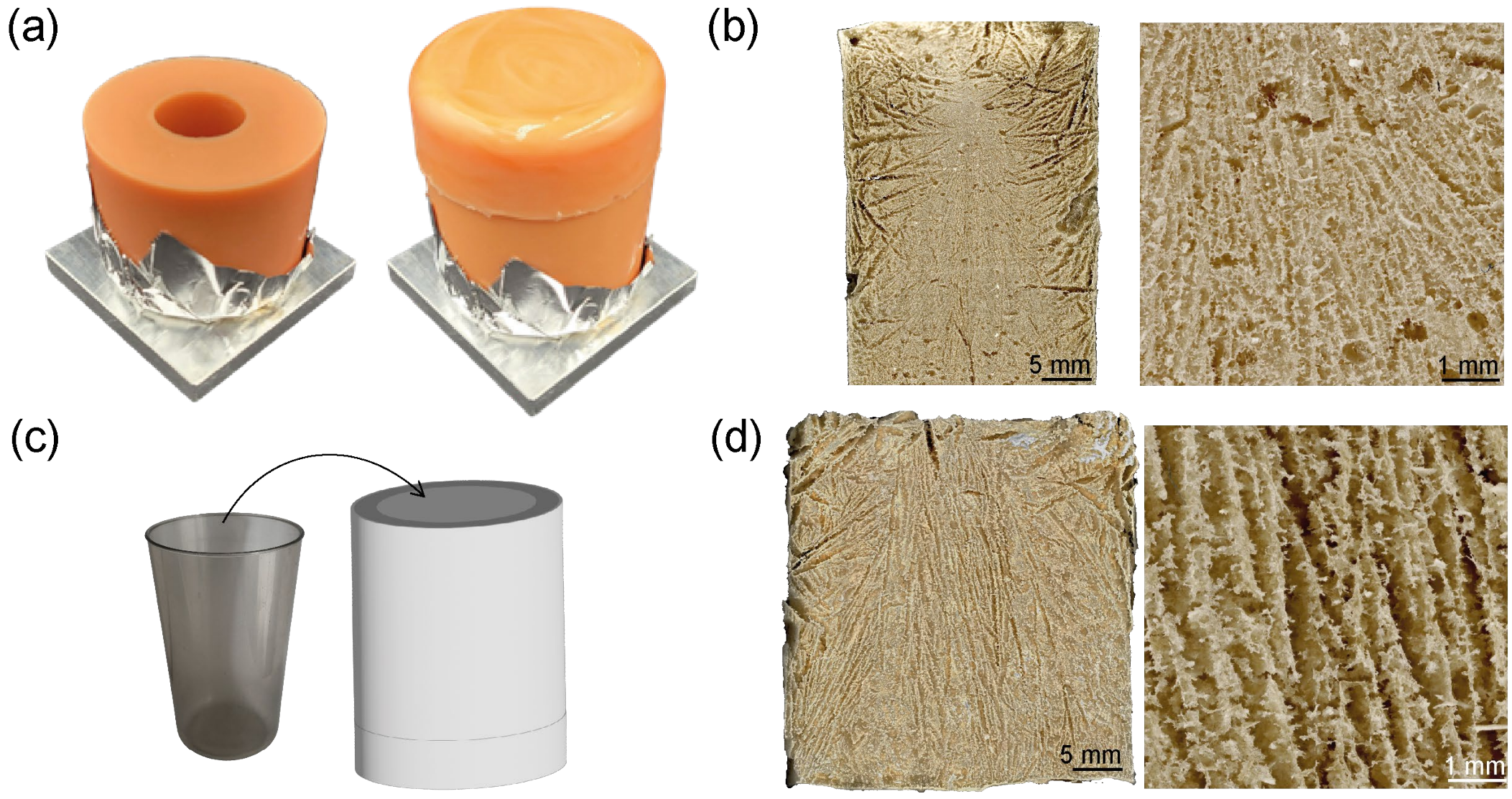

Figure S3. Freezing setups for directional freezing. (a) Silicone mould configuration: mould placed on an aluminium plate to create a bottom-up temperature gradient, without lid (before filling), then with lid in place. (b) Freeze-dried structure of a 12.5 wt% chickpea gel, frozen in the silicone mould setup. (c) Insulated beaker or container setup: plastic beaker (cylindrical or rectangular) inserted snugly into an insulating EPS foam container. (d) Freeze-dried structure of a 12.5 wt% chickpea gel prepared using the insulated beaker or container setup.

S4. Practical guide for directional freezing at home

Method to replicate directional freezing using household equipment:

1. Soak legumes: Soak whole legumes for at least 8 h in water.
2. Prepare suspension: Blend the hydrated legumes with various amounts of water to achieve the desired solids concentration (e.g., 12.5-20.0 wt%). Salt and other spices can be added freely.
3. Thermal treatment: Cook the homogenized legumes until the mixture thickens and the starch is gelatinized. This can be done:
    a. On a stovetop in a pan or pot while stirring.
    b. Using a microwave, heating in short intervals and stirring until the suspension thickens.
4. Moulding: Quickly pour the hot suspension into a plastic beaker or food-grade container.
5. Insulation setup: Place the container inside an EPS foam mould (negative) with at least 2-3 cm wall thickness. This insulation minimizes heat transfer from all sides except the top, promoting unidirectional freezing from the exposed surface downward. Do not cover the container.
6. Freezing: Put the insulated setup in a household freezer (-25 °C to -10 °C) and allow complete freezing for 12-24 hours depending on the container size.

S5. Supplementary rheological analysis

Further analyses of oscillatory shear experiments (Figure 3a) highlight differences in viscoelastic behaviour and yielding characteristics among F, DF, and R gels. Variations in G′ and G″ are most likely linked to microstructural changes induced during freezing. The loss tangent ($\tan \delta = G''/G'$) of the LVER remained relatively stable across samples (F = 0.122, DF = 0.126, R = 0.096), indicating that the elastic-viscous balance was largely unaffected. In contrast, the dynamic yield stress, derived from amplitude sweep curves, revealed marked differences: DF exhibited the highest yield stress ($\tau^* = 4.1$ kPa at 136% strain), compared to F (1.9 kPa at 130%) and R (1.6 kPa at 119%). This suggests that DF gels can withstand greater stress and deformation before yielding, consistent with their aligned lamellar structure. F formed an isotropic network with intermediate strength, whereas R showed the lowest resistance to yielding.

S6. Regression statistics for rheological analysis

Linear regression was applied to the LVER G′ (kPa) data obtained from amplitude sweep tests for F, DF, and R gels (Figure 3b). All statistical analyses were performed with Origin Pro 2025 (v10.2.0.196).

Table S2. Regression statistics for rheological analysis. Summary of linear regression parameters for LVER G′ (Pa) obtained from amplitude sweep tests. Degrees of freedom (DOF) of model and error, intercept and standard error (SE), slope and SE, Pearson correlation coefficient (Pearson R), R^2, adjusted R^2, F-value, and model significance (p-value) for chickpea gels F, DF, and R.

Sample	n	DOF	Interc.	SE	Slope	SE	Pear. R	R^2	Adj. R^2	F	p
F	7	1, 5	-14'504	4'075	3'895	303	0.99	0.97	0.96	165.63	<0.0001
DF	7	1, 5	-25'350	13'329	6'111	990	0.94	0.88	0.86	38.09	0.00163
R	7	1, 5	-21'685	5'084	3'123	377	0.97	0.93	0.92	68.40	4.22×10^{-4}

S7. Sample syneresis and corrected rheological analysis

Syneresis was determined on thawed gels by placing each sample on a 0.5 mm² mesh sieve for 3 minutes to allow liquid drainage. The drained liquid was weighed, and syneresis was calculated according to equation (S1):

$$\textit{Syneresis (\%)} = \frac{\textit{Mass of drained liquid (g)}}{\textit{Initial sample mass (g)}} \times 100 \tag{S1}$$

After syneresis, the actual solids concentration of the gel was recalculated to account for water loss according to equation (S2):

$$\textit{Corrected concentration (wt\%)} = \frac{\textit{Initial concentration (wt\%)}}{100 - \textit{Syneresis (\%)}} \times 100 \tag{S2}$$

For the 5.0 wt% R sample, syneresis reached 100%, as the sample was liquid and passed through the sieve (Figure S4a). In the rheological analysis, the entire liquid phase was measured, therefore, no syneresis correction was applied. For all other concentrations and cooling conditions, the solids were retained after syneresis and subsequently measured, therefore, concentration adjustments were applied to account for water loss (Figure S4b).

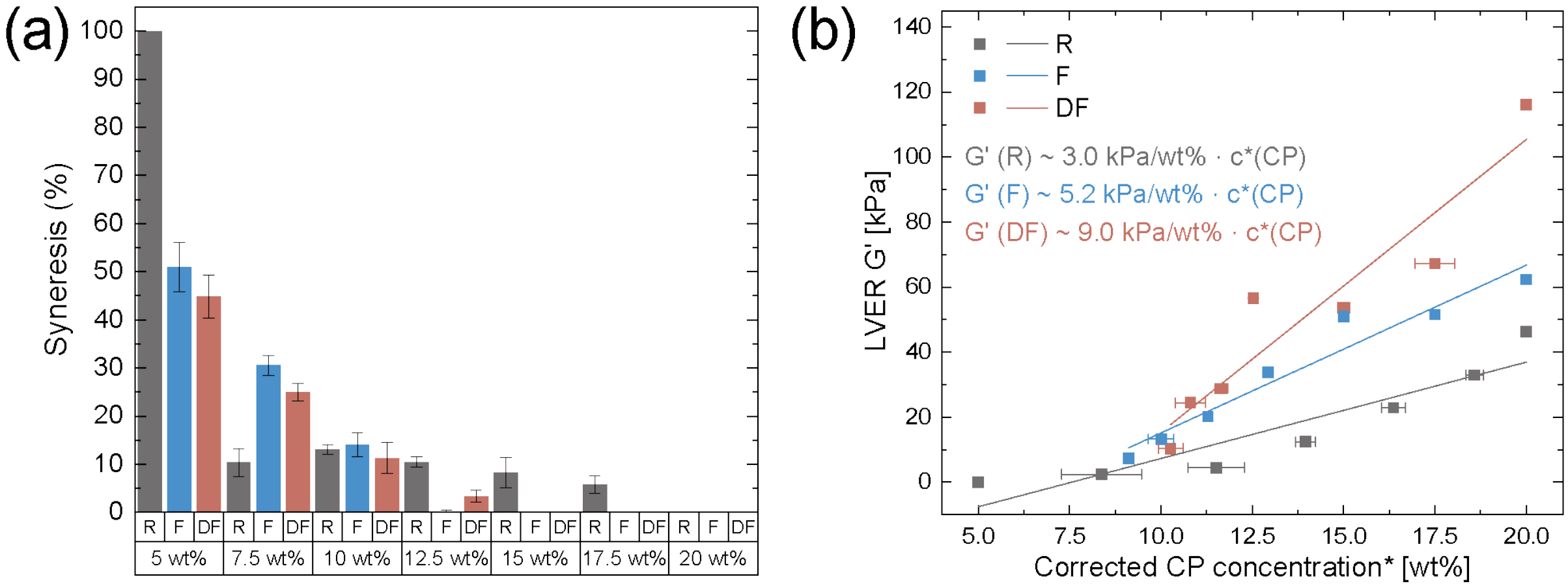

Figure S4. Syneresis and corrected rheological analysis. (a) Syneresis data for R, F, and DF chickpea gels measured at initial concentrations of 5.0 to 20 wt%. (b) Corrected rheological analysis corresponding to Figure 3b, recalculated based on actual concentrations after syneresis adjustment. G′ in the linear viscoelastic region (LVER) of amplitude sweeps of R, F, and DF gels from corrected chickpea concentrations, with corresponding regression lines.

Linear regression was applied to the LVER G′ (kPa) data obtained from amplitude sweep tests for F, DF, and R gels after correcting for actual concentrations.

Table S3. Regression statistics for rheological analysis with corrected concentrations. Summary of linear regression parameters for LVER G′ (Pa) obtained from amplitude sweep tests after correcting for actual concentrations following syneresis. Degrees of freedom (DOF) of model and error, intercept and standard error (SE),

slope and SE, Pearson correlation coefficient (Pearson R), R^2, adjusted R^2, F-value, and model significance (p-value) for chickpea gels F, DF, and R.

Sample	n	DOF	Interc.	SE	Slope	SE	Pear. R	R^2	Adj. R^2	F	p
F	7	1, 5	-22'425	7'640	2'969	535	0.93	0.86	0.83	30.97	0.00258
DF	7	1, 5	-36'490	7'972	5'162	562	0.97	0.94	0.93	84.40	2.56×10^{-4}
R	7	1, 5	-74'822	20'288	9'013	1'411	0.94	0.89	0.87	40.77	0.00140

S8. Statistics for hardness

Regression analysis of hardness measurements of 12.5-20.0 wt% chickpea gels was performed using a weighting factor of $1/e^2$ to account for measurement uncertainty (Figure S5).

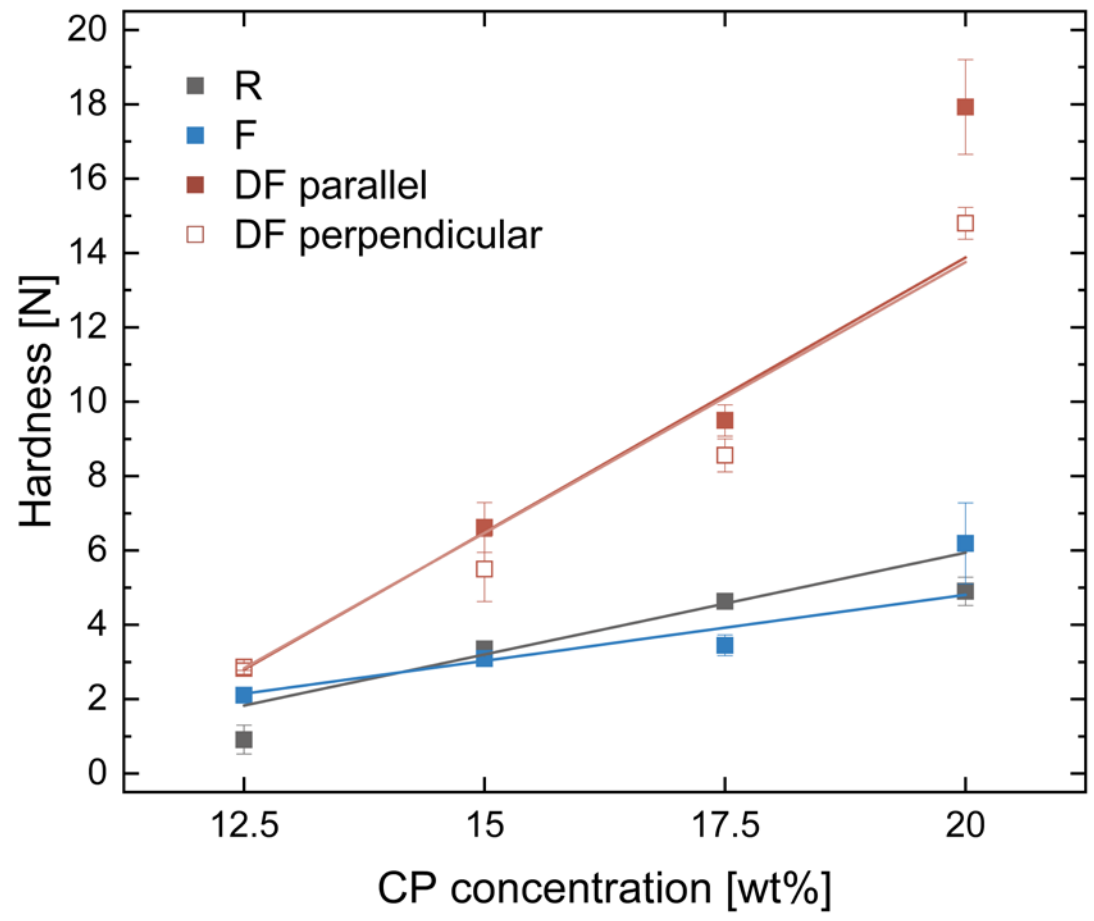

Figure S5. Hardness regression analysis for chickpea gels of 12.5 to 20.0 wt%. Hardness in N for R, F, DF parallel, and DF perpendicular chickpea samples of 12.5-20.0 wt% with corresponding regression lines.

Table S4. Regression statistics for hardness. Summary of linear regression parameters for hardness in N using a weighting of $1/e^2$ obtained from single compression tests corresponding to Figure 3d. Degrees of freedom (DOF) of model and error, intercept and standard error (SE), slope and SE, Pearson correlation coefficient (Pearson R), R^2, adjusted R^2, F-value, and model significance (p-value) for chickpea gels F, DF, and R, with DF measured in parallel (par) and perpendicular (perp) direction to the temperature gradient.

Sample	n	DOF	Interc.	SE	Slope	SE	Pear. R	R^2	Adj. R^2	F	p
DF par	4	1, 2	-0.90	0.77	3.70	0.51	0.98	0.96	0.95	53.54	0.02
DF perp	4	1, 2	-0.81	0.54	3.64	0.39	0.99	0.98	0.97	87.91	0.01
F	4	1, 2	1.26	0.32	0.89	0.17	0.96	0.93	0.89	25.75	0.04
R	4	1, 2	0.46	1.00	1.37	0.35	0.94	0.88	0.83	15.29	0.06

Comparisons were made across cooling conditions (R, F, and DF) and concentrations (12.5-20.0 wt%), and for DF samples, direction of compression (parallel vs. perpendicular). Two-way ANOVA was applied to assess the effects of cooling condition and concentration and for DF compression direction and concentration, followed by groupwise comparisons (Tukey's HSD) to identify significant differences.

Table S5. Two-Way ANOVA for hardness of chickpea gels. Summary of analysis of variance (ANOVA) results for hardness in N of R, F, and DF chickpea gels at concentrations 12.5-20.0 wt%, including their interaction. Reported values include degrees of freedom (DOF), sum of squares, mean squares, F-statistic, and model significance (p-value).

Source	DOF	Sum of squares	Mean squares	F	p
Condition	2	304.25	152.13	192.87	<0.0001
Concentration	3	314.34	104.78	132.84	<0.0001
Interaction	6	167.38	27.9	35.37	<0.0001

Model	11	957.47	87.04	110.35	<0.0001
Error	37	29.18	0.79		
Corrected Total	48	986.65			

Table S6. Groupwise comparisons for cooling conditions. Summary of mean hardness in N for chickpea gels under different conditions. Letters indicate statistically significant groupings based on Tukey's HSD post-hoc test.

Condition	Mean hardness (N)	Group
DF	8.57	A
F	3.9	B
R	3.45	B

Table S7. Groupwise comparisons for concentrations. Summary of mean hardness in N for chickpea gels at concentrations 12.5-20.0 wt%. Letters indicate statistically significant groupings based on Tukey's HSD post-hoc test.

Concentration	Mean hardness (N)	Group
20.0	10.59	A
17.5	6.53	B
15.0	4.64	C
12.5	2.18	D

Table S8. Interaction for cooling condition and concentration. Summary of mean hardness in N for chickpea gels across conditions and concentrations (12.5-20.0 wt%). Letters indicate statistically significant groupings based on Tukey's HSD post-hoc test.

Condition	Concentration	Mean hardness (N)	Group
DF	20.0	16.36	A
DF	17.5	9.03	B
F	20.0	6.19	C
DF	15.0	6.06	C
R	20.0	4.9	C D
R	17.5	4.63	C D E
F	17.5	3.45	D E
R	15.0	3.35	D E F
F	15.0	3.09	D E F
DF	12.5	2.84	D E F
F	12.5	2.11	E F
R	12.5	0.91	F

Table S9. Two-Way ANOVA for compression direction. Summary of analysis of variance (ANOVA) results for hardness in N of chickpea gels tested in parallel and perpendicular directions at concentrations 12.5-20.0 wt%. Reported values include degrees of freedom (DOF), sum of squares, mean squares, F-statistic, and model significance (p-value).

Source	DOF	Sum of squares	Mean squares	F	p
Concentration	3	600.28	200.09	466.88	<0.0001
Direction	1	9.94	9.94	23.19	0.00019
Interaction	3	7.96	2.65	6.19	0.00539
Model	7	618.18	88.31	206.06	<0.0001
Error	16	6.86	0.43		
Corrected Total	23	625.04			

Table S10. Groupwise comparison for compression direction. Summary of mean hardness in N for chickpea gels tested in parallel and perpendicular directions. Letters indicate statistically significant groupings based on Tukey's HSD post-hoc test.

Direction	Mean hardness (N)	Group
par	9.22	A
perp	7.93	B

Table S11. Interaction for concentration and compression direction. Summary of mean hardness in N for chickpea gels tested in parallel and perpendicular directions across concentrations (12.5-20.0 wt%). Letters indicate statistically significant groupings based on Tukey's HSD post-hoc test.

Concentration	Direction	Mean hardness (N)	Group
20.0	par	17.93	A
20.0	perp	14.8	B

17.5	par	9.5	C
17.5	perp	8.56	C
15.0	par	6.62	D
15.0	perp	5.5	D
12.5	perp	2.86	E
12.5	par	2.83	E

S9. Syneresis-corrected hardness

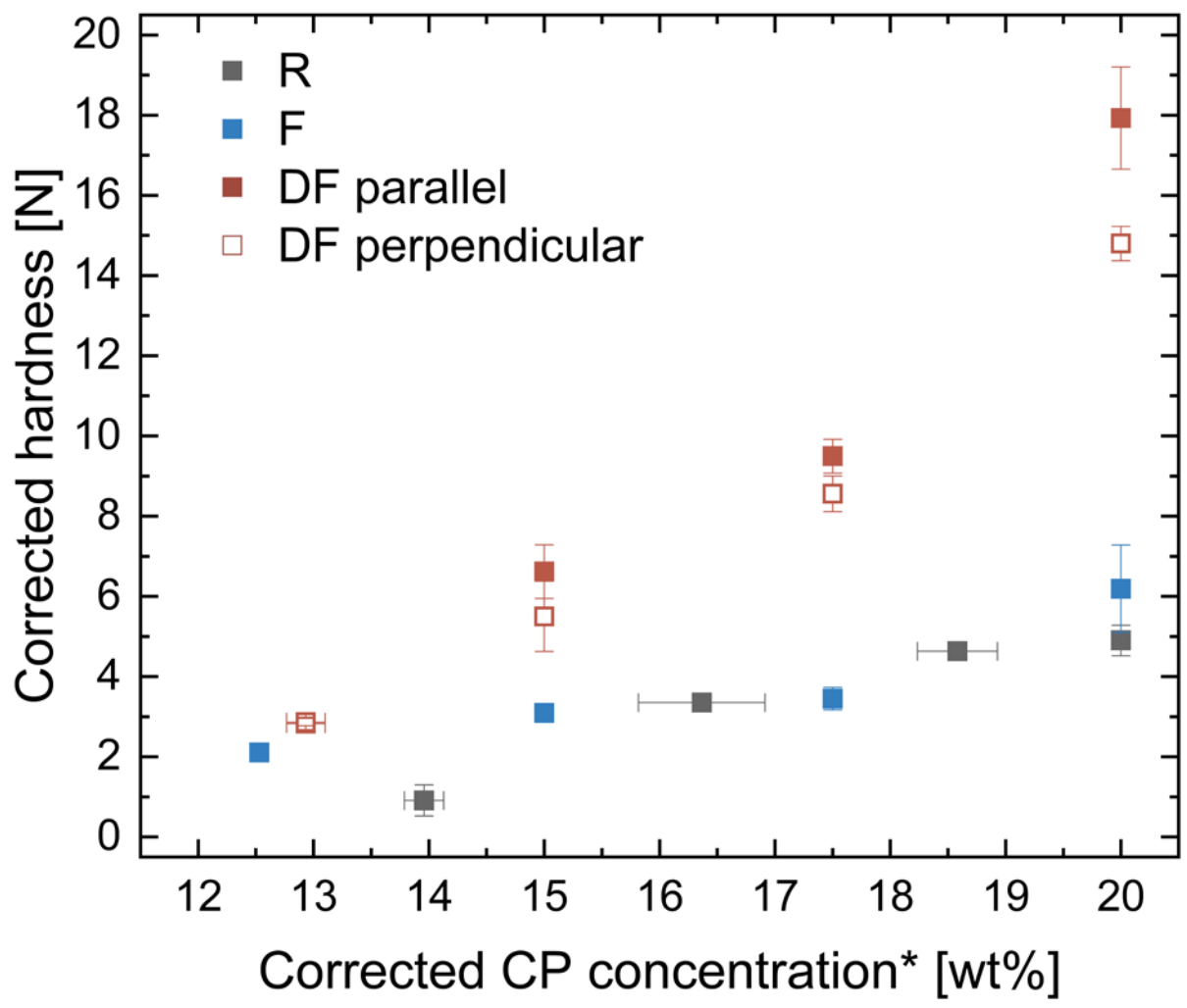

Figure S6. Hardness of chickpea gels with syneresis adjusted concentrations. Hardness of R, F, DF parallel, and DF perpendicular chickpea gels of 12.5-20.0 wt% with concentrations adjusted for syneresis.

S10. Statistics for cohesiveness

Table S12. Two-Way ANOVA for cohesiveness of chickpea gels. Summary of analysis of variance (ANOVA) results for cohesiveness (%) of R, F, and DF chickpea gels at concentrations 12.5-20.0 wt%, including their interaction. Reported values include degrees of freedom (DOF), sum of squares, mean squares, F-statistic, and model significance (p-value).

Source	DOF	Sum of squares	Mean squares	F	p
Condition	2	5714.71	2857.35	152.7	<0.0001
Concentration	3	369.02	123.01	6.57	0.00113
Interaction	6	726.05	121.01	6.47	<0.0001
Model	11	7091.54	644.69	34.45	<0.0001
Error	37	692.35	18.71		
Corrected Total	48	7783.89			

Table S13. Groupwise comparisons for cooling conditions. Summary of mean cohesiveness (%) for chickpea gels under different cooling conditions. Letters indicate statistically significant groupings based on Tukey's HSD post-hoc test.

Condition	Mean cohesiveness (%)	Group
DF	56.55	A
F	37.53	B
R	32.96	C

Table S14. Groupwise comparison for concentrations. Summary of mean cohesiveness (%) for chickpea gels at concentrations 12.5-20.0 wt%. Letters indicate statistically significant groupings based on Tukey's HSD post-hoc test.

Concentration	Mean cohesiveness (%)	Group
17.5	48.26	A
15.0	47.74	A
20.0	47.59	A
12.5	39.17	B

Table S15. Interaction for cooling condition and concentration. Summary of mean cohesiveness (%) for chickpea gels across conditions and concentrations (12.5-20.0 wt%). Letters indicate statistically significant groupings based on Tukey's HSD post-hoc test.

Condition	Concentration	Mean cohesiveness (%)	Group
DF	17.5	63.35	A
DF	15.0	59.6	A
DF	20.0	58.39	A
DF	12.5	44.86	B
F	20.0	42.3	B C
F	15.0	38.99	B C D
F	17.5	36.12	B C D
R	12.5	35.84	B C D
R	20.0	33.04	C D
R	15.0	32.78	C D
F	12.5	31.1	C D
R	17.5	30.19	D

Table S16. Two-Way ANOVA for compression direction. Summary of analysis of variance (ANOVA) results for cohesiveness (%) of chickpea gels tested in parallel and perpendicular directions at concentrations 12.5-20.0 wt%. Reported values include degrees of freedom (DOF), sum of squares, mean squares, F-statistic, and model significance (p-value).

Source	DOF	Sum of squares	Mean squares	F	p
Concentration	3	1173.28	391.09	14.05	<0.0001
Direction	1	14.06	14.06	0.51	0.4874
Interaction	3	78.56	26.19	0.94	0.444
Model	7	1265.9	180.84	6.5	0.00097
Error	16	445.25	27.83		
Corrected Total	23	1711.16			

Table S17. Groupwise comparison for compression direction. Summary of mean cohesiveness (%) for chickpea gels tested in parallel and perpendicular directions. Letters indicate statistically significant groupings based on Tukey's HSD post-hoc test.

Direction	Mean cohesiveness (%)	Group
par	57.32	A
perp	55.79	A

Table S18. Interaction for concentration and compression direction. Summary of mean cohesiveness (%) for chickpea gels tested in parallel and perpendicular directions across concentrations (12.5-20.0 wt%). Letters indicate statistically significant groupings based on Tukey's HSD post-hoc test.

Concentration	Direction	Mean cohesiveness (%)	Group
17.5	par	63.61	A
17.5	perp	63.1	A
20.0	par	61.94	A
15.0	par	60.36	A B
15.0	perp	58.84	A B
20.0	perp	54.85	A B C
12.5	perp	46.36	B C
12.5	par	43.37	C

S11. Chewiness and springiness

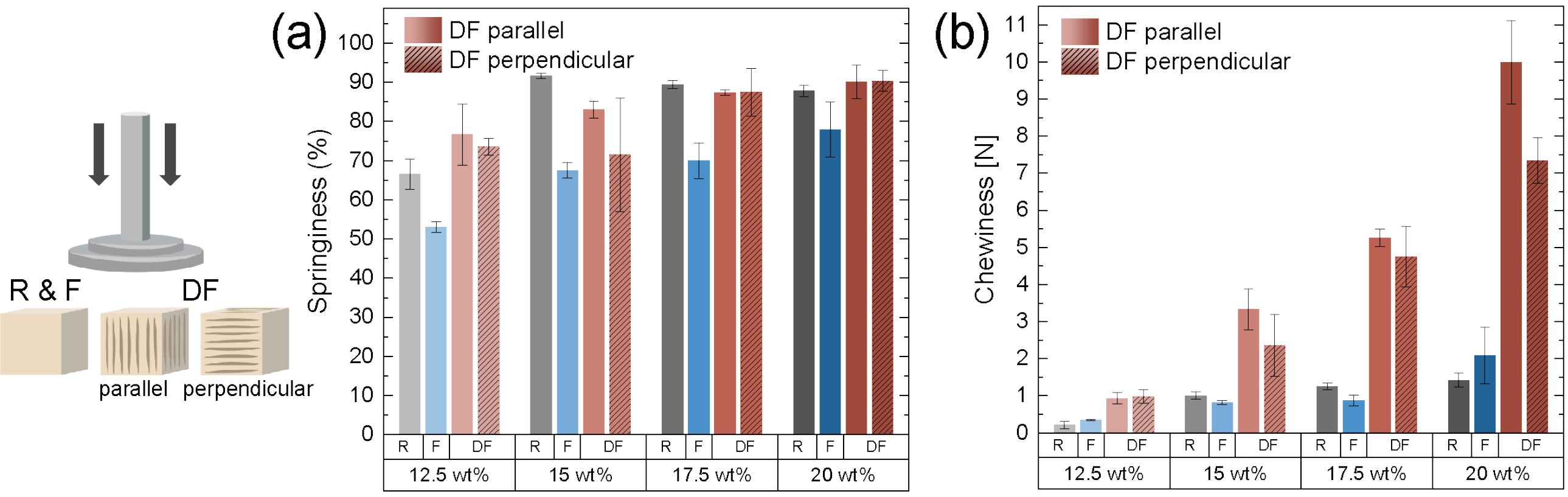

Figure S7. Springiness and chewiness of chickpea gels. Double compression of chickpea gels at concentrations 12.5-20.0 wt%, with DF measured parallel and perpendicular to the temperature gradient. (a) Springiness (%) and (b) Chewiness in N of R, F, and DF samples.

S12. Statistics for springiness

Table S19. Two-Way ANOVA for springiness (%) of chickpea gels. Summary of analysis of variance (ANOVA) results for springiness (%) of R, F, and DF chickpea gels at concentrations 12.5-20.0 wt%, including their interaction. Reported values include degrees of freedom (DOF), sum of squares, mean squares, F-statistic, and model significance (p-value).

Source	DOF	Sum of squares	Mean square	F	p
Condition	2	2377.95	1188.97	38.64	<0.0001
Concentration	3	2706.12	902.04	29.31	<0.0001
Interaction	6	660.21	110.03	3.58	0.0068
Model	11	5458.29	496.21	16.13	<0.0001
Error	37	1138.53	30.77		
Total	48	6596.82			

Table S20. Groupwise comparisons for cooling conditions. Summary of mean springiness (%) for chickpea gels under different conditions. Letters indicate statistically significant groupings based on Tukey's HSD post-hoc test.

Condition	Mean springiness (%)	Group
R	83.87	A
DF	82.52	A
F	67.98	B

Table S21. Groupwise comparisons for concentrations. Summary of mean springiness (%) for chickpea gels at concentrations 12.5-20.0 wt%. Letters indicate statistically significant groupings based on Tukey's HSD post-hoc test.

Concentration	Mean springiness (%)	Group
20.0	85.91	A
17.5	83.57	A B
15.0	78.45	B
12.5	67.46	C

Table S22. Interaction for cooling condition and concentration. Summary of mean springiness (%) for chickpea gels across conditions and concentrations (12.5-20.0 wt%). Letters indicate statistically significant groupings based on Tukey's HSD post-hoc test.

Condition	Concentration	Mean springiness (%)	Group
R	15.0	91.67	A
DF	20.0	90.22	A
R	17.5	89.42	A B
R	20.0	87.84	A B C
DF	17.5	87.44	A B
F	20.0	78.00	A B C D
DF	15.0	77.29	B C D
DF	12.5	75.13	C D
F	17.5	69.99	D
F	15.0	67.56	D E
R	12.5	66.54	D E
F	12.5	53.05	E

Table S23. Two-Way ANOVA for compression direction. Summary of analysis of variance (ANOVA) results for springiness (%) of chickpea gels tested in parallel and perpendicular directions at concentrations 12.5-20.0 wt%. Reported values include degrees of freedom (DOF), sum of squares, mean squares, F-statistic, and model significance (p-value).

Source	DOF	Sum of squares	Mean square	F	p
Concentration	3	992.34	330.78	7.70	0.0021
Direction	1	76.70	76.70	1.78	0.2003
Interaction	3	135.93	45.31	1.05	0.3960
Model	7	1204.96	172.14	4.00	0.0102
Error	16	687.77	42.99		
Total	23	1892.74			

Table S24. Groupwise comparison for compression direction. Summary of mean springiness (%) for chickpea gels tested in parallel and perpendicular directions. Letters indicate statistically significant groupings based on Tukey's HSD post-hoc test.

Concentration	Mean springiness (%)	Group
20.0	90.22	A
17.5	87.44	A B
15.0	77.29	B C
12.5	75.13	C

Table S25. Interaction for concentration and compression direction. Mean springiness (%) for chickpea gels tested in parallel and perpendicular directions across concentrations (12.5-20.0 wt%). Letters indicate statistically significant groupings based on Tukey's HSD post-hoc test.

Direction	Mean springiness (%)	Group
par	84.31	A
perp	80.73	A

S13. Statistics for chewiness

Table S26. Two-Way ANOVA for chewiness of chickpea gels. Summary of analysis of variance (ANOVA) results for chewiness of R, F, and DF chickpea gels at concentrations 12.5-20.0 wt%, including their interaction. Reported values include degrees of freedom (DOF), sum of squares, mean squares, F-statistic, and model significance (p-value).

Source	DOF	Sum of squares	Mean square	F	p
Condition	2	138.04	69.02	121.31	<0.0001
Concentration	3	77.15	25.72	45.20	<0.0001
Interaction	6	69.27	11.55	20.29	<0.0001
Model	11	340.51	30.96	54.41	<0.0001
Error	37	21.05	0.57		
Total	48	361.56			

Table S27. Groupwise comparisons for cooling conditions. Summary of mean chewiness in N for chickpea gels under different conditions. Letters indicate statistically significant groupings based on Tukey's HSD post-hoc test.

Condition	Mean chewiness (N)	Group
DF	4.37	A
F	1.11	B
R	0.98	B

Table S28. Groupwise comparisons for concentrations. Summary of mean chewiness in N for chickpea gels at concentrations 12.5-20.0 wt%. Letters indicate statistically significant groupings based on Tukey's HSD post-hoc test.

Concentration	Mean chewiness (N)	Group
20.0	4.97	A
17.5	3.03	B
15.0	1.88	C
12.5	0.62	D

Table S29. Interaction for cooling condition and concentration. Mean chewiness in N for chickpea gels across conditions and concentrations (12.5-20.0 wt%). Letters indicate statistically significant groupings based on Tukey's HSD post-hoc test.

Condition	Concentration	Mean chewiness (N)	Group
DF	20.0	8.66	A
DF	17.5	5.00	B
DF	15.0	2.85	C
RF	20.0	2.09	C D
FC	20.0	1.42	C D
FC	17.5	1.25	C D
FC	15.0	1.01	C D
DF	12.5	0.96	D
RF	17.5	0.88	D
RF	15.0	0.81	D
RF	12.5	0.35	D
FC	12.5	0.22	D

Table S30. Two-Way ANOVA for compression direction. Summary of analysis of variance (ANOVA) results for chewiness in N of chickpea gels tested in parallel and perpendicular directions at concentrations 12.5-20.0 wt%. Reported values include degrees of freedom (DOF), sum of squares, mean squares, F-statistic, and model significance (p-value).

Source	DOF	Sum of squares	Mean square	F	p
Concentration	3	196.74	65.58	154.06	<0.0001
Direction	1	6.29	6.29	14.78	0.0014
Interaction	3	6.05	2.02	4.74	0.0150
Model	7	209.08	29.87	70.17	<0.0001
Error	16	6.81	0.43		
Total	23	215.89			

Table S31. Groupwise comparison for compression direction. Summary of mean chewiness in N for chickpea gels tested in parallel and perpendicular directions. Letters indicate statistically significant groupings based on Tukey's HSD post-hoc test.

Direction	Mean chewiness (N)	Group
par	4.88	A
perp	3.86	B

Table S32. Interaction for concentration and compression direction. Summary of mean chewiness in N for chickpea gels tested in parallel and perpendicular directions across concentrations (12.5-20.0 wt%). Letters indicate statistically significant groupings based on Tukey's HSD post-hoc test.

Direction	Concentration	Mean chewiness (N)	Group
par	20.0	9.99	A
perp	20.0	7.34	B
par	17.5	5.27	C
perp	17.5	4.74	C D
par	15.0	3.33	D E
perp	15.0	2.36	E F
par	20.0	1.80	F G
par	17.5	1.06	G H
perp	12.5	0.98	F G H
par	12.5	0.94	F G H
par	15.0	0.91	G H
perp	12.5	0.28	H

S14. References for texture analysis

For texture comparison, commercially available mozzarella, brie, silken, and firm tofu were analysed. Double compression tests were performed to determine hardness, springiness, cohesiveness, and chewiness using the same experimental setup and data processing workflow as for chickpea gels (Table S32).

Table S33. Texture properties of food reference samples. Summary of texture analysis results for commercial reference samples (mozzarella, brie, silken tofu, and firm tofu) obtained using the same double compression method as for chickpea gels. Reported parameters include hardness (N), cohesiveness (%), springiness (%), and chewiness (N), with mean, standard deviation (SD), minimum, median, and maximum values (n = 3).

Property	Sample	N	Mean	SD	Minimum	Median	Maximum
**Hardness (N)**	Mozzarella	3	13.86	2.85	10.89	14.12	16.58
	Brie	3	11.81	0.64	11.13	11.88	12.42
	Silken Tofu	3	2.72	0.40	2.30	2.76	3.11
	Firm Tofu	3	31.29	0.26	31.02	31.31	31.54
**Cohesiveness**	Mozzarella	3	82.00	1.60	80.19	82.62	83.20
	Brie	3	65.78	4.23	61.27	66.43	69.64
	Silken Tofu	3	37.38	5.51	32.19	36.78	43.17
	Firm Tofu	3	70.83	1.85	69.04	70.71	72.74
**Springiness (%)**	Mozzarella	3	85.58	4.49	81.71	84.52	90.50
	Brie	3	73.21	8.06	66.41	71.11	82.11
	Silken Tofu	3	82.69	3.83	78.30	84.47	85.30
	Firm Tofu	3	91.22	4.91	87.12	89.88	96.66
**Chewiness (N)**	Mozzarella	3	9.74	2.06	7.38	10.64	10.64
	Brie	3	5.70	0.95	5.05	5.26	5.26
	Silken Tofu	3	0.85	0.26	0.66	0.75	0.75
	Firm Tofu	3	20.24	1.69	19.11	19.43	19.43

S15. Worldwide legume production

Global production volumes of major legume crops in 2023 were compiled from FAOSTAT[2]. The share of selected legumes (*) was calculated as a percentage of total legume production, representing 96% of global output (Table S33).

Table S34. Global production of major legume crops in 2023. Global production volumes of major legume crops in 2023, sorted by crop type. Data from FAOSTAT (Food and Agriculture Organization of the United Nations)[2].

Item	Value	Unit	Item Code (CPC)
Beans, dry*	28’505’529.5	t	01701
Broad beans and horse beans, dry	6’073’526.2	t	01702
Chickpeas, dry*	16’517’592.3	t	01703
Black-eyed peas, dry*	9’784’129.4	t	01706
Lentils, dry*	7’068’620.6	t	01704
Lupins*	1’918’642.2	t	01709.02
Other pulses n.e.c.	5’183’944.4	t	01709.90
Peas, dry*	13’763’334.1	t	01705
Pigeon peas, dry	4’585’466.3	t	01707
Soya beans*	371’173’609.3	t	0141
Vetches	671’463.3	t	01709.01
Selected (*)	448'731’457.5	t	
Total	465’245’857.6	t	
Percentage selected (*) of total	**96.450 %**		

S16. Additional legumes structured by directional freezing

Directional freezing was applied to a broader range of legumes to demonstrate further versatility. The following species were structured into anisotropic gels: Black lentils (BL) (*Lens culinaris),* yellow lentils (YL) (*Lens culinaris),* kidney beans (KB) (*Phaseolus vulgaris)*, navy beans (NB) (*Phaseolus vulgaris)*, and lupins (LU) (*Lupinus albus*).

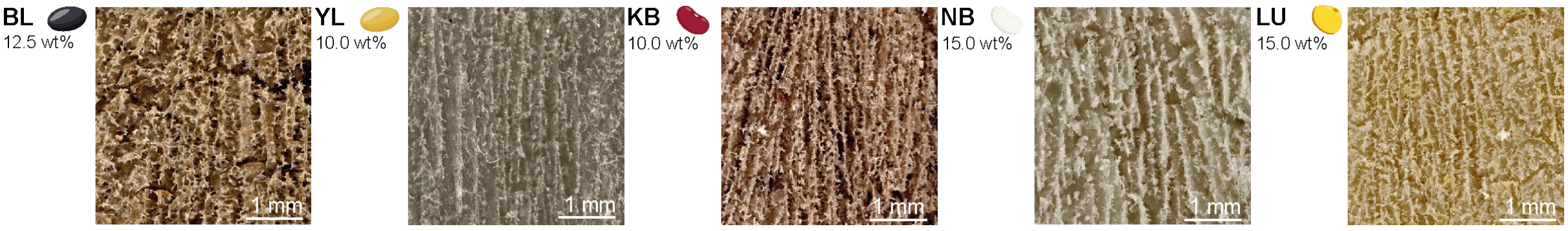

Figure S8. Additional freeze-structured legumes. Digital micrographs of freeze-dried legume gels structured under DF, including black lentil (BL, 12.5 wt%), yellow lentil (YL, 10.0 wt%), kidney bean (KB, 10.0 wt%), navy bean (NB, 15.0 wt%), and lupin (LU, 15.0 wt%).

S17. Legume composition table

Protein-to-starch ratio was calculated by dividing the protein content (in weight percent) by the starch content (in weight percent) and expressed as grams of protein per gram of starch (Table S34).

Table S35. Macronutrient composition of selected legumes. Protein, starch, fiber, and fat contents (% dry matter) and calculated protein-to-starch ratio for chickpea, red lentil, green peas, black bean, mung bean, black-eyed pea, and soybean. Data from FAO (2023)[2] and soybean values from Stevenson et al.[3].

Legume	Protein (% dry)	Non-fiber-carbohydrate (% dry)	Fiber (% dry)	Fat (% dry)	Protein/non-fiber-carbohydrate ratio (-)
Chickpea	22.4	46	22.8	4.39	0.49
Red lentil	27.1	51.7	17.1	0.95	0.52
Green peas	26.1	44.5	24	1.91	0.59
Black bean	25.1	44.8	23.8	1.55	0.56
Mung bean	23.2	54.9	17	1.16	0.42
Black-eyed pea	25.2	52.4	16.3	1.67	0.48
Soybean	38.4	20.8	4.7	18.1	1.85

S18. Okara and soy

Table S36. Macronutrient composition and partitioning during tofu production from whole soybeans. Average macronutrient composition (% dry basis) of whole soybeans, tofu, and okara, and reported average distribution of protein, lipids, and dietary fiber between tofu, okara, and whey, expressed as percentages of the total nutrient content in whole soybeans. Data adapted from van der Riet et al.[4].

	Soybeans (%)	Tofu (%)	Okara (%)	% of total in Tofu	% of total in Okara	% of total in Whey
Protein	39.8	53.15	26.9	72%	23%	8%
Lipids	19.6	32.6	10.1	82%	16%	<1%
Fibers	22.15	6.55	55.45	15%	85%	0

S19. Temperature profiles

Temperature measurements were obtained using a multichannel temperature logger equipped with thermistor sensors (Testo 176 T3, Baden-Württemberg, Germany). For the DF setup, individual sensors were embedded in silicone molds positioned along the side of the moulds at defined heights corresponding to the DF plate (0 cm), DF bottom (0.5 cm), DF middle (2.8 cm), and DF top (5.1 cm). The sensor used to record the air temperature was placed freely in the surrounding air next to the setup without enclosure. Temperature data were recorded every 15 seconds. The resulting profiles are representative curves obtained under the same freezing conditions (Figure S9).

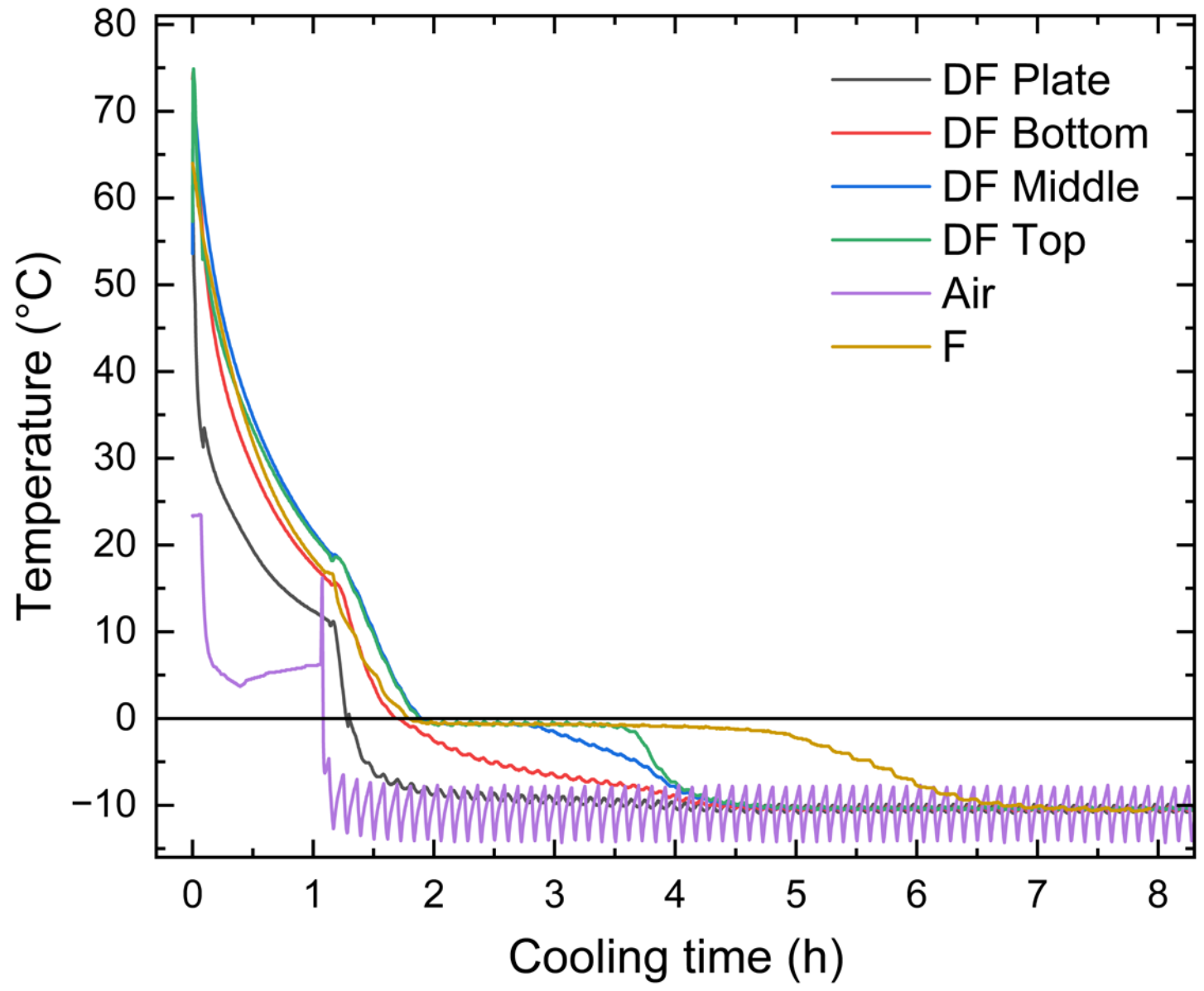

Figure S9. Temperature profiles during freezing experiments. Temperature-time profiles recorded at six positions within the directional freezing (DF) setup, including the DF plate, DF bottom, DF middle, DF top, the conventional freezing (F) setup, and the surrounding air. Data were recorded at 15-second intervals, and the displayed dataset covers the first 8 hours, corresponding to the period until equilibrium temperatures were reached. Curves are shown as continuous lines to guide the eye.

The air temperature in the shock freezer stabilized at approximately -11 °C after an initial adjustment period. Both directionally frozen (DF) and conventionally frozen (F) samples ultimately reached similar equilibrium temperatures of approximately -10 °C.

However, the cooling pathways differed markedly between the two configurations. In the DF setup, a pronounced vertical temperature gradient developed within the sample. The aluminum plate cooled most rapidly and reached subzero temperatures within approximately 1 h, followed sequentially by the DF bottom, middle, and top positions. The top of the sample crossed 0 °C only after approximately 3-4 h, demonstrating directional heat extraction from the plate upwards through the sample.

For the conventionally frozen (F) sample, the temperature recorded at the measurement position decreased more gradually, with the freezing transition occurring later (≈5–6 h). Importantly, both DF and F samples ultimately reached similar equilibrium temperatures (≈ −10 °C).

These measurements confirm that the DF configuration generates a persistent vertical temperature gradient while reaching similar final temperatures, supporting that the observed structural anisotropy arises from directional solidification rather than differences in endpoint temperature alone.

S20. References

1. Zhao, S., Ren, Y. & Wei, C. Staining starch with iodine solution. Methods Mol. Biol. 2566, 281–290 (2023).
2. FAO. FAOSTAT: Crops and livestock products. https://www.fao.org/faostat/en/#data/QCL. Licence: CC-BY-4.0 (2023).
3. Stevenson, D. G., Doorenbos, R. K., Jane, J. L. & Inglett, G. E. Structures and functional properties of starch from seeds of three soybean (Glycine max (L.) Merr.) varieties. Starch/Staerke 58, 509–519 (2006).
4. van der Riet, W. B., Wight, A. W., Cilliers, J. J. L. & Datel, J. M. Food chemical investigation of tofu and its byproduct okara. Food Chem. 34, 193–202 (1989).
